# Ligand-Enhanced Negative Images Optimized for Docking Rescoring

**DOI:** 10.3390/ijms23147871

**Published:** 2022-07-17

**Authors:** Sami T. Kurkinen, Jukka V. Lehtonen, Olli T. Pentikäinen, Pekka A. Postila

**Affiliations:** 1 Institute of Biomedicine, Integrative Physiology and Pharmacy, University of Turku, FI-20014 Turku, Finland; sami.t.kurkinen@utu.fi (S.T.K.); olli.pentikainen@utu.fi (O.T.P.); 2 Aurlide Ltd., FI-21420 Lieto, Finland; 3 InFLAMES Research Flagship Center, University of Turku, FI-20014 Turku, Finland; 4 Structural Bioinformatics Laboratory, Biochemistry, Faculty of Science and Engineering, Åbo Akademi University, FI-20500 Turku, Finland; jukka.lehtonen@abo.fi; 5 InFLAMES Research Flagship Center, Åbo Akademi University, FI-20014 Turku, Finland

**Keywords:** molecular docking, docking rescoring, virtual screening, negative image-based rescoring (R-NiB), brute-force negative image-based optimization (BR-NiB), ligand-enhanced brute-force negative image-based optimization (LBR-NiB), pharmacophore modelling

## Abstract

Despite the pivotal role of molecular docking in modern drug discovery, the default docking scoring functions often fail to recognize active ligands in virtual screening campaigns. Negative image-based rescoring improves docking enrichment by comparing the shape/electrostatic potential (ESP) of the flexible docking poses against the target protein’s inverted cavity volume. By optimizing these negative image-based (NIB) models using a greedy search, the docking rescoring yield can be improved massively and consistently. Here, a fundamental modification is implemented to this shape-focused pharmacophore modelling approach—actual ligand 3D coordinates are incorporated into the NIB models for the optimization. This hybrid approach, labelled as ligand-enhanced brute-force negative image-based optimization (LBR-NiB), takes the best from both worlds, i.e., the all-roundedness of the NIB models and the difficult to emulate atomic arrangements of actual protein-bound small-molecule ligands. Thorough benchmarking, focused on proinflammatory targets, shows that the LBR-NiB routinely improves the docking enrichment over prior iterations of the R-NiB methodology. This boost can be massive, if the added ligand information provides truly essential binding information that was lacking or completely missing from the cavity-based NIB model. On a practical level, the results indicate that the LBR-NiB typically works well when the added ligand 3D data originates from a high-quality source, such as X-ray crystallography, and, yet, the NIB model compositions can also sometimes be improved by fusing into them, for example, with flexibly docked solvent molecules. In short, the study demonstrates that the protein-bound ligands can be used to improve the shape/ESP features of the negative images for effective docking rescoring use in virtual screening.

## 1. Introduction

A multitude of rescoring and post-processing methods have been developed with case-specific success-rates for improving lackluster molecular docking yields [1,2,3,4]. Negative image-based rescoring (R-NiB) is an effective and ultra-fast way of rescoring the docking poses, using the target protein’s cavity information; furthermore, it has been shown to boost docking enrichment with various targets and docking algorithms in benchmarking [5,6,7]. In R-NiB, a negative image-based (NIB) model is generated of the protein’s ligand binding cavity using PANTHER [8], and its shape and electrostatic potential (ESP) is compared, without allowing atomic transformations against the flexibly sampled docking poses, using the similarity comparison algorithm, SHAEP [9].

Because the NIB models contain unnecessary or “extra” cavity atoms, their rescoring effectiveness can be boosted, often massively, using a systematic greedy search known as brute-force negative image-based optimization (BR-NiB) [10]. This enrichment improvement to docking can be secured, even if a limited training set containing both the known active and inactive ligands or decoys is available. During the automated greedy search, the effect of each of the positively or negatively charged (N/O) and neutral cavity atoms (C) forming the NIB model is subjected to systematic enrichment evaluation and potential exclusion from the model. Although the shape/ESP of the optimized NIB models are similarly used in the docking rescoring as with the non-optimized cavity negatives, the automatic editing or trimming transforms the R-NiB method conceptually towards the shape-focused pharmacophore modeling. The optimized NIB models should, in theory, have superior atomic compositions or shape/ESP profiles for recognizing the active ligands from decoys than the non-optimized models in the docking rescoring usage. This has been probed thoroughly via benchmarking with several datasets from the DUD (A Directory of Useful Decoys) [11], DUD-E (Database of Useful (Docking) Decoys-Enhanced) [12], DUDE-Z/Extrema [13] and MUV (Maximum Unbiased Validation) [14] databases. Whenever possible, the randomly generated training/test divisions were used, to ensure unbiased evaluation or harsh validation. Notably, BR-NiB has been shown to work—even in cross-use between different docking software, including algorithms [10] such as GLIDE [15,16], DOCK [17], GOLD [18], and PLANTS [19]. In practice, the optimization is affected by many factors, such as the quality of the flexible docking sampling, benchmarking library curation, target protein 3D structure conformation, and the user-selected target enrichment metric.

**Figure 1:**
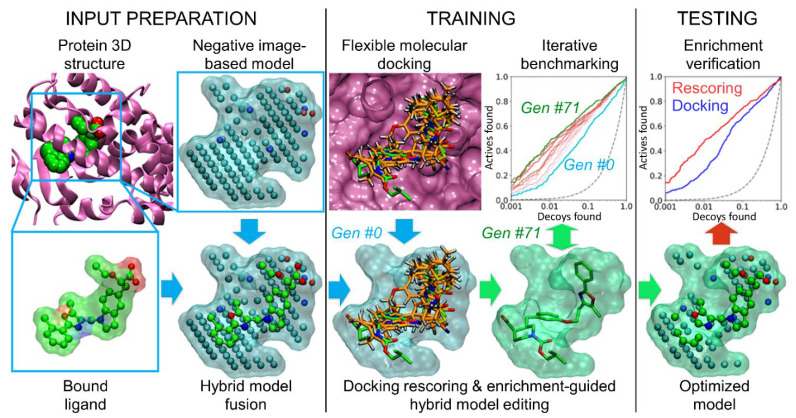
Ligand-enhanced brute-force negative image-based optimization with peroxisome proliferator-activated receptor gamma. The LBR-NiB protocol begins with input model preparation: (1) generation of the negative image-based (NIB) model of the binding pocket of PPARγ using PANTHER [8]; (2) fusing the NIB model with a protein bound (here co-crystal) ligand to form a hybrid model; (3) flexible docking of known training set of active and inactive or decoy ligands with a docking algorithm (e.g., PLANTS [19]); (4) model training or optimization of the hybrid model via iterative trimming and benchmarking with shape/ESP similarity comparison (SHAEP [9], ROCKER [20]). The successive enrichment improvements are shown between the last generation (green line; Gen #71) and first generation (cyan line; Gen #0) using semilogarithmic receiver operating characteristics (ROC) curves. The enrichment-driven greedy search optimized hybrid model contains those ligand and NIB model atoms that increase the docking rescoring yield. Before applying the optimized model in actual virtual screening, the final model can be tested using a similarly processed test set that was excluded from the model training (Table 1). The optimization cycles of LBR-NiB are identical to the BR-NiB processing, but the latter method relies solely on the cavity-based NIB models; i.e., no direct ligand 3D data is involved [10].

**Figure 2:**
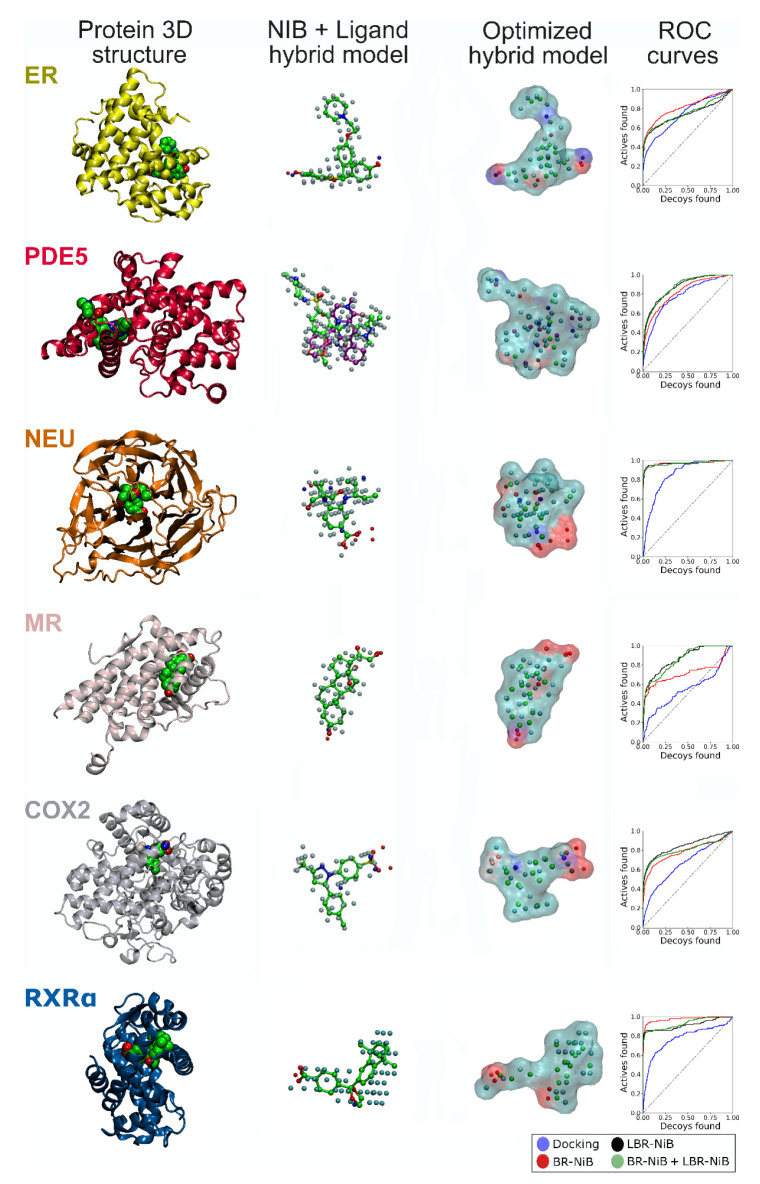
Ligand-enhanced brute-force negative image-based optimized models. From left to right: protein 3D structures for the selected DUD-E set targets (cartoon models) and the co-crystallized ligands (green CPK models), the input ligand-NIB (negative image-based) hybrid models (covalent bonds are removed), the LBR-NiB-optimized models (transparent surfaces with electrostatics coloring), and semilogarithmic ROC curves for the default docking scoring of PLANTS (blue), LBR-NiB (black), BR-NiB + LBR-NiB (green), and BR-NiB (red). The comparison of LBR-NiB against BR-NiB shows that the added ligand data can improve the very early enrichment (PDE5, COX2, MR), but, likewise, it can also weaken the overall enrichment (ER) or the already excellent BR-NiB scoring simply cannot be improved upon (NEU). The equivalent enrichment values for 100:100 training/test sets are given in Table 1 and Table 2. See Figure 1 for interpretation and PPARγ.

Likewise, the atomic composition of the original input NIB model, which provides all the shape and ESP features for the optimization, has a profound effect. Despite the intended ligand-likeness, for better or worse, the NIB models can only be perceived as pseudo-ligands, whose geometries and atomic compositions differ from those of the actual protein-bound small-molecule ligands (NIB vs. ligand; Figure 1 and Figure 2). Accordingly, the aim was to determine if the optimization process can be enhanced by incorporating the protein-bound ligand data into the cavity-based NIB models. In the ligand-enhanced brute-force negative image-based optimization (LBR-NiB; Figure 1), the cavity-based NIB models are fused with the 3D ligand coordinates and the greedy search optimization is free to select those NIB model and ligand atoms that best facilitate the recognition of the active docked ligands from the pool of decoys. The ligand atoms, acting side-by-side with the NIB model’s atoms, can originate from the ligand-protein complexes, solved for example using X-ray crystallography, nuclear magnetic resonance (NMR) spectroscopy or even from in silico sources, such as flexible molecular docking.

This hybrid approach (Figure 1) is closely related to fragment- and negative image-based (F-NiB) screening, in which the ligands are rigidly docked against the fragment-NIB hybrid models [21]. Regardless, the LBR-NiB differs from F-NiB in two important ways: (1) In F-NiB, the cavity-based NIB models are fused manually together with specifically selected ligand fragments, whereas the LBR-NiB selects the atoms from the added ligand(s) automatically and without specific user input [10]. In other words, the hybrid model crafting for effective F-NiB usage requires expertise and removal of overlapping atoms; in comparison, the atom selection process of the LBR-NiB, pioneered in this study, is fully automated; (2) In F-NiB, the ligand 3D conformers are generated ab initio for the rigid docking or F-NIB screening and, in contrast, in LBR-NiB, the ligands are docked flexibly using any standard docking algorithm (e.g., PLANTS [19]) before the model’s optimization and its final docking rescoring usage.

The LBR-NiB benchmark testing, focused here on various proinflammatory targets (Table S1), such as retinoid X receptor alpha (RXRα) [22], phosphodiesterase 5 (PDE5) [23], peroxisome proliferator-activated receptor gamma (PPARγ; Figure 1) [24] or sphingosine-1-phosphate receptor 1 (S1P1) [25], shows that the ligand 3D data can enhance the effectiveness of the NIB models in docking rescoring usage via optimization. The LBR-NiB presents a tangible advancement over the previous iterations of the negative image-based methodology in two crucial ways: (1) The LBR-NiB routinely pushes a massive improvement on the docking yield, and, while this boost is typically only slightly better than what BR-NiB generates, any improvement at the stage of very early enrichment is highly desirable for assuring a truly effective docking-based drug discovery; (2) At its best, the LBR-NiB can remedy the shortcomings of the cavity detection and filling and, consequently, improve massively the ability of the NIB models to boost docking.

Ultimately, the success of LBR-NiB over BR-NiB depends on the interplay between or combined effect of the NIB model and ligand(s) that are being fused together. However, in practice, the low-risk and potentially high-reward option is to fuse the NIB model with the co-crystal ligand taken from the original X-ray crystal structure, because its atomic arrangements are likely to be of high quality in comparison to, for example, molecular docking binding predictions. In conclusion, LBR-NiB typically poses very little extra computing costs over BR-NiB; in fact, the ligand data can even be incorporated into a previously BR-NiB-optimized NIB model—this dual processing approach generated the most consistent boost to docking in the benchmark testing.

## 2. Results and Discussion

### 2.1. Fusion of Ligands into the Cavity-Based Negative Images

LBR-NiB (Figure 1) is a direct modification of the established BR-NiB methodology presented in a prior study [10]. Between the methods, the only difference is the atomic compositions of the respective input models. BR-NiB is performed using the cavity-based NIB models, whereas LBR-NiB relies on the ligand-NIB hybrid models. During the ligand and NIB model fusion (Figure 1 and Figure 2), the ligand’s covalent bonding information is removed and its potential atomic overlaps with the NIB model atoms are ignored. There are two fundamental differences between these two types of input models at the atomic level. Firstly, all of the atoms of the protein-bound ligands contain partial charge information, whereas the NIB models typically contain partially positive (N) and negative (O) atoms that designate H-bond donors and acceptors, respectively, as well as neutral filler atoms (C). Secondly, the hybrid model generation introduces a diverse set of ligand atoms with varying atomic radii that can be better or worse in describing the cavity volume than the three-component arrangement.

#### 2.1.1. Ligands Boost Performance of Negative Image-Based Models via Optimization

Without the optimization, the ligand-NIB hybrid models produced mixed results in the docking rescoring usage (Tables S2 and S3, Supplementary Materials). This can be seen both without the training/test set division (Gen #0: 100:100 in Table S2, Supplementary Materials), and with the two applied ratios of 70:30 and 10:90 (Gen #0: Tables S2 and S3, Supplementary Materials). Although the hybrid models could occasionally improve on the default docking scoring (e.g., MR in Tables S2 and S3, Supplementary Materials), typically the enrichment was weakened by applying the hybrid model in rescoring (e.g., PDE5 or PPARγ; Tables S2 and S3, Supplementary Materials). Likewise, rescoring with the hybrid models did not usually generate a higher enrichment than the standard R-NiB; regardless, the AUC values could still be at a statistical tie in this match-up (Tables S2 and S3, Supplementary Materials).

In contrast, after the LBR-NiB processing, the co-crystal ligand-NIB hybrid models could work well or excellently in rescoring use (Figure 1 and Figure 2; Table 1 and Table S4, Supplementary Materials). The LBR-NiB outperformed the original docking with all targets and with all of the applied training/test set ratios, notably, the AUC values of ER and PPARγ remained at a statistical tie with the original docking (Table 1), which likely reflects the mixed agonist/antagonist composition of these two DUD-E datasets. Importantly, the hybrid models could generate higher EFd 1% values with any training/test set ratio than BR-NiB with COX2, PDE5 and, especially, with PPARγ (Table S5, Supplementary Materials). The added ligand atoms weakened the ER results most consistently against BR-NiB in the testing, regardless of the applied training/test set ratio (Figure 2; Table S5, Supplementary Materials). When utilizing the shape-only option in this match-up, the LBR-NiB could outperform BR-NiB, especially with 100:100 and 10:90 ratios (e.g., NEU; Table S5, Supplementary Materials).

LBR-NiB routinely boosted the default docking performance, as was to be expected, based on the prior BR-NiB results [10]. However, the practice of adding the atomic data from the co-crystallized ligands into the NIB models for the optimization (Figure 1) was not always beneficial in comparison to BR-NiB (e.g., ER in Table S5, Supplementary Materials). An obvious fix is to incorporate the different co-crystallized ligands into the NIB models and, for example with RXRα, the results improved markedly by incorporating a different ligand (HXA to BM6; BM6-based model in Figure 2; Table 1; Table S5, Supplementary Materials), although BR-NiB could still outperform LBR-NiB with the 70:30 and 10:90 sets (Table S5, Supplementary Materials). When the LBR-NiB did improve on the BR-NiB enrichment, the level of the docking boost varied between moderate and excellent (Table S5, Supplementary Materials). The highest increase was recorded for PPARγ, the EFd 1% value of BR-NiB was improved 21.1-, 13.7- or 23.9-percentage points by the LBR-NiB treatment for the 100:100, 70:30 or 10:90 test sets, respectively (50/50 shape/ESP in Table S5, Supplementary Materials).

#### 2.1.2. Two Ligands for Boosting Phosphodiesterase 5 Enrichment

PDE5 is a difficult target for flexible docking or docking rescoring using the R-NiB method, based on benchmarking [5,7]. The known PDE5 inhibitors that are included in the DUD-E set belong in several camps: for example, sildenafil-like and tadalafil-like compounds have completely different binding modes. These two inhibitor subgroups occupy only partially overlapping parts of the spacious binding cavity of PDE5. Accordingly, it is difficult to directly generate a cavity-based NIB model that would be able to filter a mix of these inhibitors from the pool of “inactive” decoys (Table S1, Supplementary Materials). However, the rescoring could succeed at least in the benchmarking settings, if two separate NIB models were generated to match roughly the dimensions of either one of these protein-bound co-crystallized inhibitors, before their combined BR-NiB processing [10].

By also adding the atoms of the co-crystallized ligands into the two-cavity NIB model, the BR-NiB results were surpassed by LBR-NiB for PDE5 with all of the applied training/test set ratios (Figure 2; Table 1; Table S5, Supplementary Materials). This impressive enrichment improvement over the regular BR-NiB was seen not only with the target metric BR20, but also with EFd 1%, EFd 5% and AUC values with statistical significance. In comparison to the prior BR-NiB results for PDE5, the EFd 1% values of LBR-NiB were improved 8.5, 2.5- or 11.4-percentage points by the LBR-NiB treatment for the 100:100, 70:30 or 10:90 test sets, respectively (50/50 shape/ESP weight; Table S5, Supplementary Materials). While this elaborate protocol works for PDE5 based on the enrichment gains (Table 1; Figure 2), inarguably, it would be preferable to perform BR-NiB or LBR-NiB using a training set that does not present opposing demands on the model being optimized.

The extra cost of adding two or more ligands, simultaneously, into the input NIB model for hybrid optimization could be worth considering on a routine basis. This multi-ligand approach makes most sense when BR-NiB or single-ligand LBR-NiB is not working well enough, and the NIB model or training set is not prohibitively enormous.

#### 2.1.3. Consistent Boost by Fusing Ligands into the Pre-Optimized Negative Image-Based Models

The BR-NiB-optimized NIB models perform excellently in the docking rescoring, using randomly generated training/test set divisions [10], although they retain less atomic data than the non-optimized input NIB models (red lines in Figure 2). In fact, including the co-crystallized ligand atoms for the optimization did not boost performance in all of the test cases, as detailed above (e.g., ER in Table 1 and Table S5, Supplementary Materials; black lines in Figure 2). However, because BR-NiB was performed in the prior study using the same training/test sets [10], the co-crystallized ligands (Table S1, Supplementary Materials) could also be incorporated into these pre-optimized NIB models for another round of the LBR-NiB processing (Table 2; Table S6, Supplementary Materials; green lines in Figure 2).

This minor change to the LBR-NiB protocol did not necessarily assure the best enrichment (Table 1 vs. Table 2); in fact, the two-step approach generated typically lower enrichment gains than the one-step LBR-NiB approach (Table S5 vs. Table S7, Supplementary Materials). However, in return, the dual optimization method provided a consistent boost over the prior BR-NiB processing (Table 2 and Table S7, Supplementary Materials). The early enrichment factors and even the AUC values of BR-NiB were improved consistently for most of the targets using the applied training/test set ratios (BR-NiB + LBR-NiB in Table 2). The only notable exceptions were the LBR-NiB-optimized models for RXRα and ER that could fare worse than their BR-NiB-optimized counterparts, for example, with the 100:100 and 10:90 ratios using the equal shape/ESP weight.

The stepwise or dual optimization protocol, applying the pre-optimized NIB models as a basis for the ligand fusion, is less likely to provide massive gains than the all-in LBR-NiB approach but it is also more likely to improve the results (Table S5 vs. Table S7, Supplementary Materials). Of course, if the additional LBR-NiB step does not improve on the BR-NiB yields, the user can simply revert to using the original BR-NiB-optimized model instead (e.g., RXRα; Table 2).

### 2.2. Dataset Curation Is Crucial for the Optimization

LBR-NiB was also tested using all of the MUV [14] sets with X-ray crystal structures containing co-crystal ligands (Table S1, Supplementary Materials). These were already similarly probed with the BR-NiB method in a prior study [10]. The MUV database is more suitable to benchmark ligand-based screening methods than molecular docking because it contains an extremely limited assortment of different active ligand chemotypes for each target. In addition, due to the low compound numbers, no training/test set divisions could be made in the same way as was completed with the DUD-E sets (100:100 in Table S1, Supplementary Materials). This harsh difficulty for docking is reflected as low enrichment values generated by the original docking scoring; for example, the AUC values ranged from 0.32 ± 0.04 to 0.54 ± 0.05 (Table S8, Supplementary Materials).

Both the BR-NiB and LBR-NiB were able to improve on the default docking enrichment with all of the MUV targets (Tables S8 and S9, Supplementary Materials). In many cases, the use of the only shape scoring in the optimization generated better results than the regular 50/50 shape/ESP scoring; this was especially visible with EFd 0.5% and/or EFd 1%. However, the LBR-NiB often performed worse than the BR-NiB, regardless of the applied shape/ESP weight, and it could outperform the BR-NiB only occasionally regarding the very early enrichment. The LBR-NiB was prominently effective only for Cathepsin G (CathG) for which the EFd 0.5% improved 10-percentage points in comparison to BR-NiB. In contrast, the LBR-NiB performed noticeably weaker than BR-NiB with sphingosine-1-phosphate receptor 1 and coagulation factor XIa (FXIa). The results were mixed when fusing the ligands into the pre-optimized NIB models and, particularly, EFd 0.5% could be improved sometimes (e.g., human immunodeficiency virus reverse transcriptase RNase). On the other hand, for example, FXIa failed totally when using the 50/50 shape/ESP scoring in the optimization. The AUC values of BR-NiB could not be improved statistically for the MUV sets with the LBR-NiB method.

Even though the MUV datasets are very demanding for molecular docking, the BR-NiB was able to boost the docking results (Table S9, Supplementary Materials) [10]. The LBR-NiB did not improve on the BR-NiB results overall, which suggests that the specificity of the added ligand atoms limited the all-rounded performance of the NIB models. In other words, the LBR-NiB-optimized models were more focused than the BR-NiB-optimized models—this bias is especially detrimental to the enrichment metrics when dealing with the MUV sets that contain a tiny amount of extremely diverse active ligands (Table S1, Supplementary Materials).

### 2.3. Docked Solvent Boosts the Negative Image-Based Models Performance via Optimization

**Figure 3:**
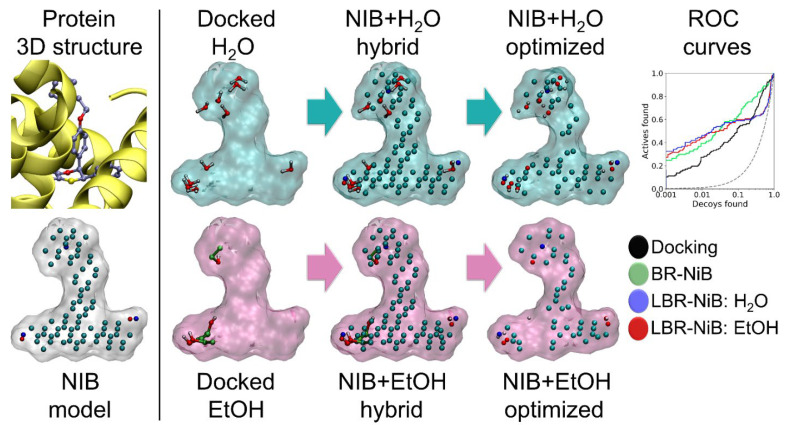
Solvent-enhanced brute-force negative image-based optimization with estrogen receptor alpha. Water (H_2_O) and ethanol (EtOH) molecules were docked flexibly into the ligand-binding cavity of estrogen receptor (ER). The docked solvent molecules were combined with the PANTHER-generated NIB model, bonding information was removed, and the resulting solvent-NIB hybrid models were optimized similarly, as was completed with hybrid models incorporating the co-crystallized ligands (Figure 1 and Figure 2). The ROC curves show that the addition of H_2_O (green line) or EtOH (red line) provided better rescoring enrichment than flexible docking (black line) or BR-NiB (blue line), which relied only on the cavity-based NIB model. The equivalent enrichment values are given in Table 3. See Figure 1 for interpretation.

Due to the inherent flexibility of the LBR-NiB method (Figure 1), there are no strict limitations on what type of small-molecule ligands can be fused with the NIB model for the optimization. Water and ethanol, which were docked flexibly into the cavities of six DUD-E targets (Table S1, Supplementary Materials), were chosen as probes for potentially improving the NIB model compositions, especially regarding H-bonding hotspots (see ER in Figure 3). As a rule of thumb, even the most hydrophobic cavity must contain at least water, however disordered, if the cavity indeed exists [10]. Thus, in theory, water is an ideal solvent for probing the H-bonding hotspots inside cavities, due to its dual acceptor/donor role. Ethanol is also an enticing probe for matching both the hydrophobic and polar features of protein cavities, due to its amphipathic profile.

The water-NIB hybrid models, trained with the 70:30 sets (Table S10, Supplementary Materials), generated higher enrichment than the default docking scoring (Table 3). The water molecules provided a minor improvement for the LBR-NiB over BR-NiB in most cases; even the AUC values were boosted. Regardless, the water-enhanced BR-NiB could not improve the EFd 1% values of MR and COX2 or the AUC value of ER. Likewise, the incorporation of docked ethanol molecules into the NIB models could either improve (e.g., NEU and ER) or weaken (e.g., RXRα and MR) the rescoring performance over BR-NiB. Applying the shape-only option in the LBR-NiB could also be boosted by incorporating solvent molecules into the NIB models for the optimization. Although the addition of the solvent could work, the enrichment gains were limited in comparison to the standard BR-NiB.

The docked solvent can boost BR-NiB performance (e.g., ER in Figure 3)—to a certain extent this is likely to be the case with any docked small-molecules—but these positive effects are case-specific and depend on the original NIB model’s shortcomings or downright errors.

### 2.4. Fused Ligands Reduce Chemotypic Diversity

In the ligand-based screening, the alternative ligand conformers originating from a vast screening database are superimposed against a ligand 3D template, based on their shape/ESP similarity [26,27]. The method, that typically relies on protein-bound co-crystallized ligand(s), works effectively in practical drug discovery and, moreover, the template ligands can even be used directly in docking rescoring similarly to how it is executed with the NIB models. The BR/LBR-NiB-optimized models perform better than any single co-crystallized ligand in the rescoring usage, based on benchmarking (Table S11, Supplementary Materials) [10]. It is also likely that the added ligand 3D data introduce bias to the compound selection towards the compounds with similar chemotypes, thus, potentially lowering the novelty of the ligands that can be discovered in actual virtual screening assay work.

Indeed, the similarity analysis, that was performed using Tanimoto fingerprinting (Table S12, Supplementary Materials), suggests that the incorporated ligand atoms lower the diversity at the top 5% of the ranking list. Firstly, the co-crystallized ligands added into the models (Figure 2; Table S1, Supplementary Materials) were similar in comparison to all of the active ligands included in the studied DUD-E sets; the similarity ranged from 0.024 to 0.124. Secondly, the similarity with the co-crystallized ligands increased consistently for the top 5% of active compounds ranked using the BR-NiB or LBR-NiB methods. The exceptions to this rule were NEU and PDE5. With PDE5, the comparison had to rely on either tadalafil or sildenafil, instead of the two-ligand set-up used in the greedy search optimization. Thirdly, when comparing the BR-NiB to LBR-NiB, the latter method acquired typically higher similarity values than the former, which suggests that the added ligand atoms biased the sorting towards similar training set entries as the co-crystal ligand. The minor exceptions to this trend included PPARγ and NEU, or the separate PDE5 entries discussed above.

The differences in the Tanimoto scores between the top 5% results from BR-NiB and LBR-NiB were small (Table S12, Supplementary Materials). The biggest difference was seen with MR (0.111 vs. 0.308), but RXRα (0.193 vs. 0.253) and ER (0.182 vs. 0.289) also showed some bias towards the co-crystallized ligands. That is not to say that the BR-NiB and LBR-NiB-optimized models were necessarily selecting completely different active ligands at the top of their respective ranking lists. When focusing on the 5% of the top-ranked actives, the biggest differences in the top compound picks were seen for MR, NEU and RXRα (100%), although the ligand set sizes were limited for these targets (Table S1, Supplementary Materials). However, when focusing on the 25% of the top-ranked actives, the differences were higher (e.g., NEU 60%, MR 58%, PPARγ 59%).

The Tanimoto analysis (Table S12, Supplementary Materials) shows that the BR-NiB processing lowers the chemotypic diversity at the top, which is a sign that the optimized NIB models are focusing the compound selection to certain active ligand chemotypes. The addition of ligand data into the NIB models for the LBR-NiB processing introduce a further similarity bias; however, based on the numbers, this effect is not alarming.

### 2.5. Effect of Fused Ligands on the Negative Image-Based Model Performance

It is not always easy to generate a NIB model using PANTHER that contains all of the key features needed for effective docking rescoring. If the input model is lacking some key shape/ESP features to begin with, the BR-NiB simply cannot trim the model to perfection. By introducing the ligand atoms either after the initial round of optimization (BR-NiB + LBR-NiB; Table 2) or in-one-go (LBR-NiB; Figure 1; Table 1), the new atomic content is provided to the iterative trimming and evaluation process. Importantly, the direct incorporation of the ligand data into the input model pushes the methodology, for better or worse, closer to the ligand-based drug discovery methodology from the opposing camp of structure-based drug discovery (Figure 1).

**Figure 4:**
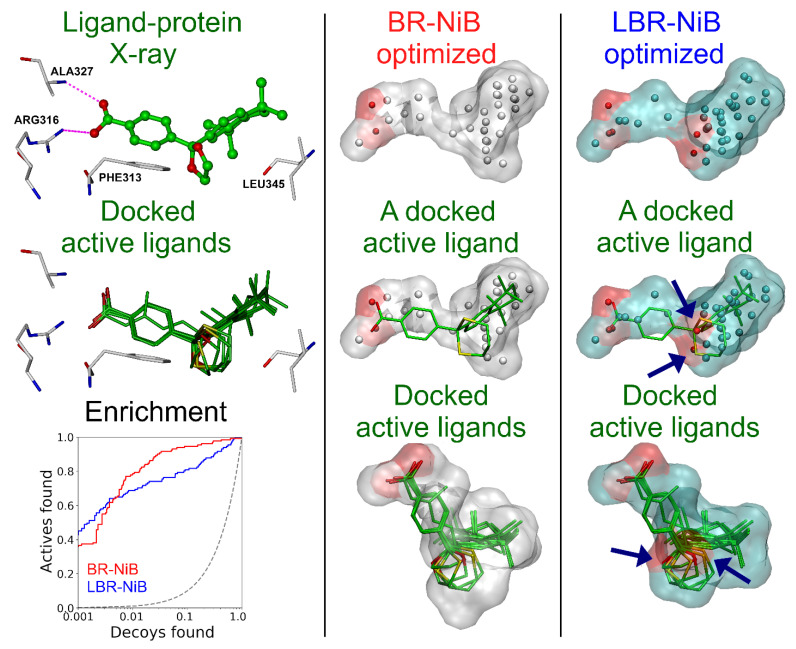
The effect of ligand-derived atoms on the docking enrichment with retinoid X receptor alpha. BR-NiB generated an effective NIB model that encompasses key shape/ESP features of RXRα binding cavity needed for docking rescoring. The incorporation of co-crystal ligand atoms (BM6 in PDB: 1MVC; ball-and-stick model) into the input NIB model generates a highly similar model after LBR-NiB processing, however, two extra polar atoms are taken from the ligand’s five-membered ring. These oxygen atoms align well with similar atoms present in a subset of the active ligands (six green stick models; e.g., CHEMBL113864) ranked at the top (blue arrows). In contrast, similarly high shape/ESP similarity match cannot be acquired for the BR-NiB-optimized model and these same docked active ligands. The polar atoms are missing from the PANTHER-generated NIB model because the ligand’s oxygen atoms are not acting as H-bond acceptors (magenta dotted lines). This ligand-derived feature seems to provide higher very early enrichment for LBR-NiB (blue line) than BR-NiB (red line), but, at the same time, it also lowers the model’s overall rescoring fitness—a trend that is clearly visible in the semilogarithmic ROC curves (Table 1). See Figure 1 for interpretation.

This methodological shift has both its pros and cons. The benefit is found in improved and very early enrichment in comparison to BR-NiB with most of the targets (Tables S5 and S7, Supplementary Materials); however, the drawback is that the model is more prone to get stuck on non-essential or non-binding related chemotypic features present in the co-crystal ligand and the training set. In other words, the ligand might insert specific atomic arrangements that improve early enrichment during rescoring, but these new features might not be necessarily helpful for filtering other active ligand chemotypes. For example, the most notable difference between the BR-NiB and LBR-NiB-optimized models for RXRα is the presence of new polar ring atoms in the latter (BM6 in Figure 4). Notably, these two oxygen atoms, which are not forming direct interactions, such as H-bonds with the protein (BM6 in Figure 4), align well with a subset of the docked active ligands with similar polar ring systems. This ligand-derived ring content improves the very early enrichment of docking marginally in comparison to BR-NiB, however, the overall yield is weakened. This suggests that the ligand data force the NIB model to specialize in ligands with similar ring systems, but it is not a helpful feature on a more general level.

**Figure 5:**
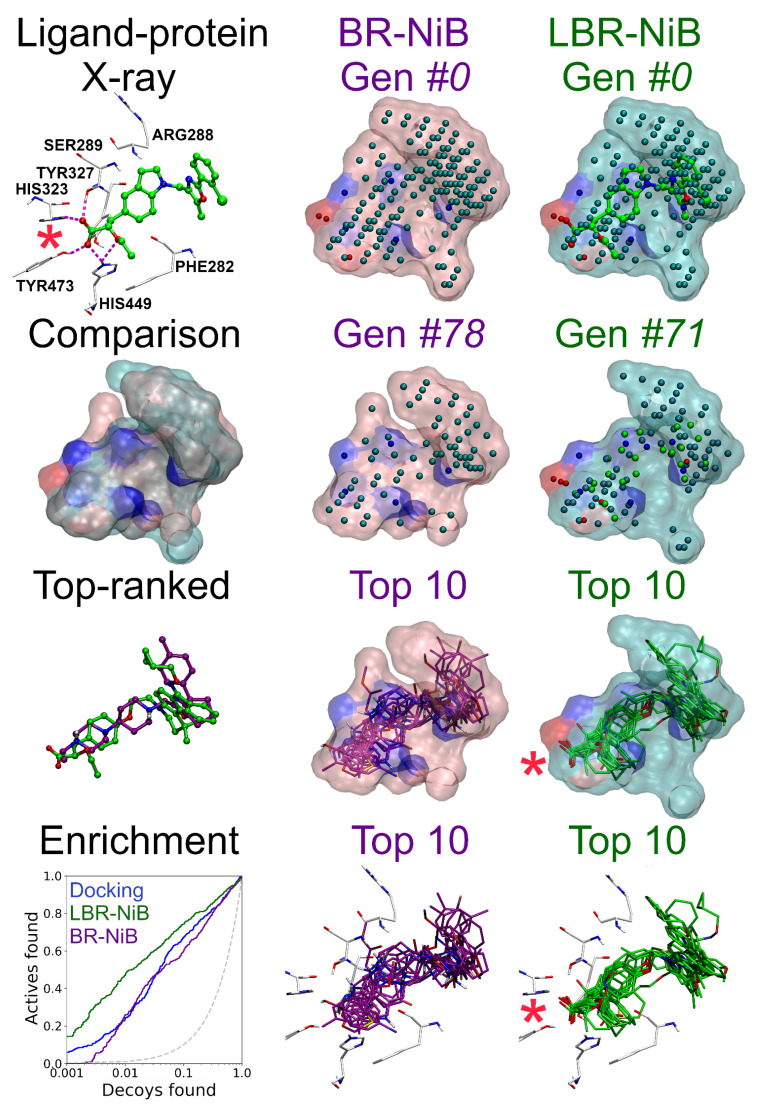
Ligand-derived atoms improve acid-containing active ligand recognition for peroxisome proliferator-activated receptor gamma. First row, agonist small-molecule ligand (compound **208**; green ball-and-stick model) bound to the PPARγ protein 3D structure (PDB: 2GTK) [28] with key binding site residues shown (white stick models); NIB (negative image-based) model of the ligand-binding pocket (Gen #0; pink transparent surface); and ligand-NIB hybrid model (Gen #0; cyan transparent surface). Second row, the comparison of LBR-NiB and BR-NiB optimized models; the BR-NiB-optimized NIB model (Gen #78); and the LBR-NiB-optimized hybrid model (Gen #71). Third row, the top-ranked compound for BR-NiB is a decoy (magenta stick model) and an active ligand for LBR-NiB (CHEMBL537921; green stick model); overlay of top 10 BR-NiB-ranked compounds (all decoys) shown against the optimized NIB model; and overlay of top 10 LBR-NiB-ranked ligands (all actives) shown against the optimized hybrid model. Fourth row, the semilogarithmic ROC curves for BR-NiB (magenta), LBR-NiB (green) and default docking scoring of PLANTS (blue); the same top-ranked ligands for BR-NiB and LBR-NiB with the key binding cavity residues shown. The red asterisks (*) pinpoint the pivotal acid placement needed for assuring top-ranking for the acid containing PPARγ active ligands during rescoring. See Figure 1 and Figure 5 for interpretation.

When the LBR-NiB boost is substantial on every level, it is easier to see how the added ligand data are behind the positive change (see PPARγ in Figure 5). With PPARγ, neither R-NiB or BR-NiB is performing well, as the docking results could worsen or the enrichment gains remain minor during rescoring, respectively [5,10]. Accordingly, the top-ranked compounds from the BR-NiB were decoys (magenta sticks in Figure 4) and the opposite was true for the LBR-NiB that selected active molecules to the top of the ranking list (green sticks in Figure 5) and improved on the default docking as well (Table 1). All of the top-ranked active ligands contained a carboxylate group acquiring canonical 3D positioning at the PPARγ cavity that is reminiscent of the acid placement of the co-crystal ligand (red asterisk in Figure 5). Although the original NIB model contained charged cavity atoms at the same whereabouts, their positioning was not spot-on and, moreover, the filler atoms close-by were masking their intended function as H-bond acceptors. As a result, the BR-NiB focused on the shape factor and trimmed these polar atoms, in contrast, the LBR-NiB retained them and ranked the acid-containing active ligands at the top.

In practice, if the PANTHER-generated NIB model is already covering all of the key sections of the cavity or ligand-binding hot-spots, the LBR-NiB is less likely to provide a substantial boost over the BR-NiB. A smaller scale boost over the BR-NiB is, however, highly likely, but it can come with unwanted chemotypic bias or baggage that the user must be aware of.

### 2.6. Practical Considerations of Using the Greedy Search Optimization

The BR-NiB or LBR-NiB are greedy search-driven methods i.e., they focus squarely on improving the model’s shape/ESP features in the sorting of docked active ligands from the decoys. Therefore, one should pay attention to two crucial factors during their usage. Firstly, the training set should be curated to contain active ligands with the same mode of action. When dealing with too diverse sets (e.g., the MUV set in Table S8, Supplementary Materials), there is a risk that a substantial number of compounds require reciprocal induced-fit effects upon binding, or that they even bind to entirely different binding sites. Secondly, one should make sure that the docking software can recreate the experimentally validated ligand-binding poses. This is not straightforward, as limited amounts of the co-crystallized ligands exist for comparison, moreover, it is not always clear if the known pose is biologically relevant or if there might actually exist multiple valid poses [29].

The RMSD calculations, comparing docking poses against the co-crystallized poses, can be problematic, as even tiny and irrelevant shifts can ruin the score. For example, the analysis suggests that the docking failed miserably for the PPARγ (Table S13, Supplementary Materials), but the top ligands, as ranked by the LBR-NiB, were reasonably aligned regarding the key acid group placement (red asterisk in Figure 5). Nevertheless, based on the limited RMSD analysis for the co-crystallized DUD-E ligands, the LBR-NiB does not steer the binding pose selection more towards “wrong” poses than the default docking scoring or the standard BR-NiB. In fact, when focusing on the 1 Å range, LBR-NiB selected the correct poses better than BR-NiB (70.6% vs. 58.8% in Table S13, Supplementary Materials) or the default docking scoring (70.6% vs. 47.1% in Table S13, Supplementary Materials).

The BR-NiB or LBR-NiB user should evaluate the top screening results carefully, and preferably post-process them, using, for example, pharmacophore filtering, redocking with alternative docking software or binding free energy calculations. However, the fact that both optimization methods worked well using the random training/test set divisions (10:90; 70:30 in Table S1, Supplementary Materials) is a strong signal that the optimization is not prone to overfitting the models to an alarming degree. It is also reassuring that the BR-NiB-optimized models could be cross-used between the docking software, suggesting that the final models contain shape/ESP-based sorting power that is rooted to a wide selection of active and decoy compounds [10].

## 3. Materials and Methods

The flexible molecular docking, negative image-based (NIB) model generation, co-crystal ligand preparation, ligand-NIB model fusion and hybrid model optimization were performed at the same location, i.e., without allowing 3D coordinate transformations (Figure 1). Moreover, to keep the results comparable with prior studies, the software settings, training/test set divisions and models described below were kept the same as before [7,10]. The new input ligand-NIB hybrid models and optimized models used in the optimization are included in the Supporting Information (SI).

### 3.1. Protein Structure Preparation

The protein 3D structures (Figure 2; Table S1, Supplementary Materials) for cyclooxygenase 2 (COX2), retinoid X receptor alpha (RXRα), mineralocorticoid receptor (MR), neuraminidase (NEU), phosphodiesterase 5 (PDE5), estrogen receptor alpha (ER) and peroxisome proliferator-activated receptor gamma (PPARγ; Figure 1) were acquired from the Protein Data Bank (PDB; www.rcsb.org, accessed on 7 December 2019 [30]). The REDUCE3.24 [31] was used for protonating the structures at pH 7.4, and necessary editing, such as PDB conversion to SYBYL MOL2 format and extraction of the co-crystallized ligand was completed in the BODIL Modelling Environment [32]. Similar preparation was also completed for the target structures of MUV database if a valid crystal structure containing a co-crystallized ligand was available. This included sphingosine-1-phosphate receptor 1 (S1P1), protein kinase A (PKA), Rho-associated protein kinase 2 (Rho-K2), human immunodeficiency virus reverse transcriptase RNase (HIV), heat shock protein 90 (HSP90), focal adhesion kinase (FAK), Cathepsin G (Cath G), coagulation factor XIa (FXIa) and coagulation factor XIIa (FXIIa).

### 3.2. Ligand Structure Preparation

The ligand test sets (Table S1, Supplementary Materials) were downloaded from the DUD-E [12] and MUV [14] databases [7,10]. The SMILES-to-MOL2 conversion, protonation at pH 7.4, tautomerization and insertion of OPLS3 partial charges were completed using LIGPREP and MOL2CONVERT in MAESTRO 2017-1 (Schrödinger, LLC, New York, NY, USA, 2017). The random 70:30 and 10:90 training/test set divisions were generated using standard C++ library Mersenne Twister 19,937, pseudorandom number generator [33]. Before inserting the co-crystallized ligands (Table S1, Supplementary Materials) into the cavity-based NIB models (Figure 1 and Figure 2), the OPLS3 force field [34] partial charges were added without allowing a coordinate shift, using FFLD_SERVER in MAESTRO; the ligands were deprotonated and converted from MAE to MOL2 format with MOL2CONVERT.

### 3.3. Flexible Molecular Docking

The role of flexible docking in the LBR-NiB or BR-NiB is to generate alternative ligand 3D conformers, for comparing against the target protein’s inverted ligand binding cavity. The flexible ligand molecular docking was completed in prior studies [5,7,10], with PLANTS1.2 [19] using the co-crystallized ligand (Figure 1 and Figure 2; Table S1, Supplementary Materials) centroid as the center for docking, with search radius of 10 Å. The flexible docking was completed using the default settings. A total of 10 alternative poses were output for each compound. To test the effect of adding explicit solvent molecules into the NIB models, water or ethanol molecules were docked in the binding cavity using the same centroid.

### 3.4. Ligand-Negative Hybrid Model Generation

The negative image-based (NIB; Figure 1 and Figure 2) models of the target protein ligand binding pockets were prepared using PANTHER0.18.15 (http://www.medchem.fi/panther/, accessed on 9 June 2022) [8]. In most cases, the NIB models were generated using the ligand distance limit option (1.5–2.0 Å) that prevents the model from expanding too far from the volume occupied by the co-crystallized or docked ligand. The centroid of a co-crystallized ligand was used as a cavity centroid, and the used box radius varied between 20–27 Å. The target-specific cavity detection settings are explained in depth in prior studies [7,10]. The hybrid models were generated by fusing together the deprotonated ligands containing OPLS3 partial charges (added using FFLD_SERVER in MAESTRO 2017-1) with the cavity-based NIB models (Figure 1 and Figure 2; Table S1, Supplementary Materials). No atoms were removed during the fusion, i.e., the atomic overlaps were allowed, and all of the covalent bonding information was removed.

In the solvent-NIB hybrid model approach, the utilized solvent molecules that were clearly outside the original NIB model volume were excluded and, furthermore, only the polar hydrogens were retained. Altogether, 10 water molecules and five ethanol molecules were used in building these hybrid models.

### 3.5. Negative Image-Based Rescoring

In the negative image-based rescoring (R-NiB) [5], the shape and electrostatic potential (ESP) of the NIB model is compared to flexible docking poses, using the similarity comparison algorithm, SHAEP [9] (Figure 1). As the docking poses and the NIB model are already in the same 3D coordinates located in the binding cavity of the target protein, no geometric optimization is performed during the similarity comparison (*--noOptimization*). The higher the similarity score, the better the docking pose matches the shape/ESP of the template model. Thus, in theory, if the NIB model is of a correct composition, the compounds with the best SHAEP scores should be active, and their binding poses should match the key features of target cavity. Here, the comparison scoring was performed using either equal shape/ESP (50/50) weight or relying on the shape only (100/0). R-NiB can be performed using either the PANTHER-generated NIB models or the ligand-NIB hybrid models.

### 3.6. Brute Force Negative Image-Based Optimization

In brute-force negative image-based optimization (BR-NiB), the input model fitness for rescoring usage is improved systematically by removing those atoms that lower the target metric enrichment with a greedy search [10]. Although ideal for solving problems with the optimal substructure, greedy approaches have been implemented successfully, for example, in docking sampling and, likewise, they were shown to perform well in NIB model optimization [10,17,35]. Ligand-enhanced brute-force negative image-based optimization (LBR-NiB; Figure 1) does the same as BR-NiB (https://github.com/jvlehtonen/brutenib, accessed on 9 June 2022), but instead of utilizing the PANTHER-generated NIB models as input, the optimization is performed on the ligand-NIB hybrid models (Figure 1 and Figure 2). The iterative atom removals continue for as long as the docking yield continues to improve in the benchmarking. A set of active and decoy ligands are used as a training set, to evaluate the model’s ability to separate active ligands from the decoy compounds with each test case after the trimming. The similarity comparison between the hybrid model and the docked training set is performed with SHAEP, in the same way as with R-NiB.

ROCKER0.1.4 [20] (http://www.medchem.fi/rocker/, accessed on 9 June 2022) is used to calculate the user-selected target enrichment metric. The Boltzmann-Enhanced Discrimination of the Receiver Operating Characteristic (BEDROC [36]) with an alpha value of 20 (referred here as BR20) was selected as the target metric. A different target metric could be more successful on a case-by-case basis, but the BR20 was used to keep the results comparable with the prior study presenting the BR-NiB method [10]. In practice, the LBR-NiB removes each cavity atom one at a time and calculates the BR20 value for each (−1 cavity atom) model. If any of the new model variants generates better BR20 value than the other models, it is selected for the next round of atom removals/benchmarking, and the cycle, spanning potentially multiple generations, starts anew. The approach does not consider every permutation of the model atom compositions (nor bonding), yet this greedy search approach has been shown to be remarkably effective with the cavity-based NIB models in benchmarking [10].

### 3.7. Data Analysis and Figure Preparation

ROCKER 0.1.4 [20] was used to calculate the enrichment metrics: the area under the curve (AUC), BR20 and early enrichment factors, i.e., the percentage of active molecules that are ranked higher than 1% or 5% of the decoy molecules (EFd 1% and EFd 5%). ROCKER was also used to plot the linear and semilogarithmic receiver operating characteristics (ROC) curves. Fingerprints and Tanimoto similarity were calculated with CANVAS in MAESTRO 2021-4 (Schrödinger, LLC, New York, NY, USA, 2021), using 32-bit precision and Daylight invariant atom types. The Root-Mean-Square Deviation (RMSD) values were calculated for those DUD-E compounds with both the docking poses and co-crystallized ligand 3D conformers, using rmsd.py (MAESTRO 2018-1). The conformers were aligned in the 3D space, using the protein backbone C^α^ atoms with VERTAA in BODIL. SDFCONF [37] was used for the handling, filtering and grouping of the docked compounds during the data analysis. The figures were prepared using BODIL, MAESTRO and VMD1.9.2 [32,38].

## 4. Conclusions

Negative image-based rescoring (R-NiB) improves the docking enrichment via shape/ESP similarity comparison of the target’s inverted cavity volume or the negative image-based (NIB) model against the flexibly sampled docking poses. Although the R-NiB yields can be improved massively using brute-force negative image-based optimization (BR-NiB) [10], the NIB models are not actual small-molecule ligands regarding their atomic compositions or geometries. Here, a new methodological modification—ligand-enhanced brute-force negative image-based optimization (LBR-NiB; Figure 1)—is presented for remedying this issue, by fusing the key atomic content of protein-bound ligands into the NIB models via a greedy search. The ligand-binding poses used in the hybrid model generation were taken directly from the ligand-protein complexes solved using X-ray crystallography (Figure 1 and Figure 2; Table S1, Supplementary Materials), but the use of docked solvent molecules was also probed (Figure 3; Table 3). The LBR-NiB improves massively on the enrichment of the default docking scoring and R-NiB, but its gains over standard BR-NiB are only occasionally substantial (Figure 4) if the original NIB model generation had not failed on a fundamental level (Figure 5). Regardless, the LBR-NiB often excels over BR-NiB on the very early enrichment, which is a crucial factor for the real-world drug discovery work, dealing with massive compound databases. Because the ligands are moderately sized in comparison to the cavity-based NIB models, their inclusion into the optimization process only marginally increases the workload. In practice, the LBR-NIB results show that the co-crystallized ligands should be incorporated into the NIB models prior to the optimization on a routine basis to assure the best docking rescoring yields and, if not available, one should consider adding other docked small-molecules, such as solvent. In conclusion, the study demonstrates that protein-bound ligand atomic data can augment the shape/ESP characteristics of the cavity negatives via greedy search optimization to facilitate effective docking-based virtual screening.

## Figures and Tables

**Table 1:** Ligand-enhanced brute-force negative image-based optimization using models combined with co-crystallized ligands in testing.

Train/ Test ^(1)^	Method ^(2)^	Yield	COX2 + CEL	RXRα + HXA	RXRα + BM6	MR + AS4	NEU + RA2	PDE5 + VIA/CIA	ER + E4D	PPARγ + 208
100:100	LBR-NiB	AUC	0.85 ± 0.01	0.92 ± 0.02	0.91 ± 0.02	0.86 ± 0.02	0.97 ± 0.01	0.87 ± 0.01	0.74 ± 0.01 (↔)	0.88 ± 0.01
		EFd 1%	44.8	65.7	78.6	27.7	81.6	28.6	35.8	46.9
		EFd 5%	63.5	77.1	85.5	57.5	92.9	53.8	51.7	65.9
		BR20	0.61	0.77	0.84	0.51	0.90	0.48	0.50	0.63
	LBR-NiB + shape only	AUC	0.85 ± 0.01	0.90 ± 0.02	0.93 ± 0.02	0.81 ± 0.03	0.97 ± 0.01	0.88 ± 0.01	0.67 ± 0.02 (↓)	0.85 ± 0.01 (↔)
		EFd 1%	50.6	67.2	69.5	30.9	81.6	29.4	36.3	42.4
		EFd 5%	64.4	77.9	84.7	48.9	93.9	53.3	45.2	59.5
		BR20	0.63	0.76	0.82	0.47	0.90	0.49	0.46	0.58
70:30	LBR-NiB	AUC	0.84 ± 0.02	0.93 ± 0.03	0.94 ± 0.03	0.84 ± 0.05	0.95 ± 0.03	0.90 ± 0.02	0.77 ± 0.03 (↔)	0.86 ± 0.02 (↔)
		EFd 1%	43.0	70.0	72.5	31.0	70.0	17.5	37.6	42.5
		EFd 5%	61.5	80.0	87.5	55.2	86.7	55.0	49.6	63.7
		BR20	0.60	0.81	0.84	0.53	0.82	0.46	0.51	0.61
	LBR-NiB + shape only	AUC	0.83 ± 0.02	0.93 ± 0.03	0.92 ± 0.03	0.82 ± 0.05	0.94 ± 0.03	0.90 ± 0.02	0.74 ± 0.03 (↔)	0.84 ± 0.02 (↔)
		EFd 1%	31.1	30.0	72.5	31.0	70.0	27.5	38.5	38.4
		EFd 5%	59.3	65.0	87.5	48.3	80.0	54.2	47.0	56.2
		BR20	0.53	0.59	0.83	0.47	0.82	0.49	0.49	0.55
10:90	LBR-NiB	AUC	0.83 ± 0.01	0.91 ± 0.02	0.91 ± 0.02	0.76 ± 0.03	0.93 ± 0.02	0.83 ± 0.01	0.69 ± 0.02 (↔)	0.86 ± 0.01 (↔)
		EFd 1%	35.2	41.5	56.8	20.0	48.3	16.7	29.1	35.8
		EFd 5%	57.9	58.5	75.4	36.5	73.0	38.2	40.9	57.8
		BR20	0.55	0.60	0.71	0.36	0.68	0.36	0.42	0.55
	LBR-NiB + shape only	AUC	0.83 ± 0.01	0.71 ± 0.03	0.88 ± 0.02	0.78 ± 0.03	0.94 ± 0.02	0.83 ± 0.01	0.73 ± 0.02 (↔)	0.84 ± 0.01 (↔)
		EFd 1%	45.2	21.2	61.0	21.2	50.6	17.8	28.8	25.9
		EFd 5%	59.7	36.4 (↓)	76.3	38.8	71.9	37.9	39.5	51.8 (↓)
		BR20	0.59	0.39	0.73	0.37	0.69	0.36	0.40	0.48 (↓)

The values are shown with downward (↓) or level (↔) arrows, if decreased or equal in comparison to default docking scoring [10]. The values are underlined, if equal or improved in comparison to the equivalent BR-NIB results (i.e., no 3D ligand data included) [10]. ^(1)^ Training/test set ratios (100:100, 70:30, 10:90): the percentage of ligands used in the training (100%, 70%, 10%) in relation to the percentage used in the testing (100%, 30%, 90%); ^(2)^ Methods: flexible docking and LBR-NiB either with the equal shape/ESP (0.5/0.5) weight or the shape only (1.0/0.0).

**Table 2:** Ligand-enhanced brute-force negative image-based optimization of preoptimized cavity models combined with co-crystallized ligands in testing.

Train/ Test ^(1)^	Method ^(2)^	Yield	COX2 + CEL	RXRα + HXA	RXRα + BM6	MR + AS4	NEU + RA2	PDE5 + VIA/CIA	ER + E4D	PPARγ + 208
100:100	BR-NiB + LBR-NiB	AUC	0.80 ± 0.01	***0.92 ± 0.02***	***0.93 ± 0.02***	***0.85 ± 0.02***	***0.97 ± 0.01***	***0.87 ± 0.01***	***0.75 ± 0.02* (↔)**	***0.87 ± 0.01* (↔)**
		EFd 1%	43.0	***70.2***	75.6	***30.9***	***85.7***	25.4	***39.2***	38.4
		EFd 5%	59.5	***80.9***	84.0	54.3	91.8	50.8	***52.0***	61.4
		BR20	0.59	***0.79***	0.83	***0.51***	***0.90***	0.46	***0.52***	0.58
	BR-NiB + LBR-NiB + shape only	AUC	***0.85 ± 0.01***	0.90 ± 0.02	***0.91 ± 0.02***	***0.80 ± 0.03***	0.97 ± 0.01	***0.88 ± 0.01***	***0.68 ± 0.02* (↓)**	***0.84 ± 0.01* (↔)**
		EFd 1%	50.3	65.6	***73.3***	***31.9***	83.7	31.4	***37.1***	***43.0***
		EFd 5%	63.7	***78.6***	83.2	45.7	91.8	***55.3***	***47.3***	58.9
		BR20	0.63	***0.76***	***0.82***	0.46	0.90	***0.50***	***0.47***	***0.58***
70:30	BR-NiB + LBR-NiB	AUC	0.78 ± 0.02	***0.93 ± 0.03***	***0.98 ± 0.01***	***0.86 ± 0.04***	***0.93 ± 0.03***	***0.88 ± 0.02***	***0.80 ± 0.02***	***0.86 ± 0.02*** **(↔)**
		EFd 1%	***48.1***	***70.0***	***80.0***	***31.0***	***63.3***	***20.0***	30.8	37.0
		EFd 5%	56.3	***85.0***	85.0	***55.2***	***83.3***	48.3	46.2	62.3
		BR20	0.58	***0.82***	***0.84***	***0.54***	0.78	0.43	0.46	0.57
	BR-NiB + LBR-NiB + shape only	AUC	***0.84 ± 0.02***	***0.90 ± 0.03***	***0.92 ± 0.03***	***0.82 ± 0.05***	***0.94 ± 0.03***	**0.90 ± 0.02**	***0.72 ± 0.03***	***0.84 ± 0.02* (↔)**
		EFd 1%	***46.7***	***70.0***	***75.0***	20.1	***83.3***	***20.8***	38.5	***40.4***
		EFd 5%	***61.5***	***80.0***	***87.5***	***48.3***	***93.3***	50.8	***49.6***	54.8
		BR20	***0.61***	***0.77***	***0.84***	0.46	0.81	0.46	***0.50***	***0.56***
10:90	BR-NiB + LBR-NiB	AUC	***0.83 ± 0.01***	***0.89 ± 0.02***	***0.88 ± 0.02***	***0.82 ± 0.09***	***0.96 ± 0.01***	***0.83 ± 0.01***	***0.68 ± 0.02***	***0.88 ± 0.01***
		EFd 1%	32.4	48.3	***65.3***	***23.9***	***64.9***	***19.2***	***29.1***	33.9
		EFd 5%	57.1	***67.8***	73.7	***39.8***	***85.1***	***40.5***	***42.1***	***62.4***
		BR20	0.53	***0.66***	***0.75***	***0.39***	***0.80***	***0.37***	***0.42***	***0.56***
	BR-NiB + LBR-NiB + shape only	AUC	***0.83 ± 0.01***	***0.80 ± 0.02*** **(↔)**	***0.86 ± 0.02***	***0.77 ± 0.03***	***0.95 ± 0.02***	***0.84 ± 0.01***	***0.73 ± 0.02***	***0.85 ± 0.01***
		EFd 1%	***46.7***	***39.8***	55.9	***22.7***	***61.7***	17.4	29.8	***28.3***
		EFd 5%	57.9	***52.5***	71.2	***39.8***	***81.9***	***41.3***	***45.1***	***53.2*** **(↓)**
		BR20	***0.59***	***0.53***	0.70	***0.38***	***0.78***	***0.38***	***0.45***	***0.50***

The values are shown with downward (↓) or level (↔) arrows, if decreased or equal in comparison to default docking scoring [10]. The values are underlined, if equal or improved in comparison to the equivalent BR-NIB results (i.e., no 3D ligand data included) [10]. Values are shown in bold and italics, if improved or equal to LBR-NiB performed with the co-crystallized ligands and non-optimized NIB models (Table 1). ^(1)^ Training/test set ratios (100:100, 70:30, 10:90): the percentage of ligands used in the training (100%, 70%, 10%) in relation to the percentage used in the testing (100%, 30%, 90%); ^(2)^ Methods: flexible docking and LBR-NiB either with the equal shape/ESP (0.5/0.5) weight or the shape only (1.0/0.0).

**Table 3:** Ligand-enhanced brute-force negative image-based optimization with cavity models combined with docked solvent in testing.

Train/ Test ^(1)^	Method ^(2)^	Yield	COX2	RXRα	MR	NEU	PDE5	ER	PPARγ
water 70:30	LBR-NiB	AUC	0.78 ± 0.01	0.95 ± 0.02	0.73 ± 0.05	0.93 ± 0.03	0.83 ± 0.02	0.72 ± 0.03 (↔)	0.84 ± 0.02 (↔)
		EFd 1%	29.1	***82.5***	17.2	***73.3***	15.8	***46.2***	38.4
		EFd 5%	51.0	***89.0***	44.8	86.7	38.3	***57.3***	56.2 (↓)
		BR20	0.48	***0.87***	0.39	0.81	0.35	***0.57***	0.54
	LBR-NiB + shape only	AUC	0.83 ± 0.02	0.90 ± 0.03	0.70 ± 0.05	0.94 ± 0.03	0.89 ± 0.02	0.73 ± 0.03 (↔)	0.82 ± 0.02 (↔)
		EFd 1%	***40.7***	***67.5***	24.1	66.7	23.3	***44.4***	***41.1***
		EFd 5%	58.5	***80.0***	48.3	***86.7***	47.5	***49.6***	52.7 (↓)
		BR20	**0.58**	***0.77***	0.42	0.82	0.46	***0.52***	0.54
ethanol 70:30	LBR-NiB	AUC	0.79 ± 0.02	0.97 ± 0.02	0.77 ± 0.05	0.94 ± 0.03	0.82 ± 0.02	0.72 ± 0.03 (↔)	0.82 ± 0.02 (↔)
		EFd 1%	38.5	***77.5***	31.0	***73.3***	*18.3*	***43.6***	32.9
		EFd 5%	50.4	***87.5***	44.8	86.7	39.2	***53.0***	47.9 (↓)
		BR20	0.54	***0.86***	0.45	***0.83***	0.36	***0.55***	0.47 (↓)
	LBR-NiB + shape only	AUC	0.82 ± 0.02	0.90 ± 0.03	0.79 ± 0.05	0.94 ± 0.03	0.88 ± 0.02	0.71 ± 0.03 (↓)	0.84 ± 0.02 (↔)
		EFd 1%	**43.7**	***45.0***	27.6	60.0	19.2	33.3	37.7
		EFd 5%	***60.0***	***85.0***	48.3	***83.3***	45.8	***49.6***	52.7 (↓)
		BR20	***0.59***	***0.74***	0.45	0.80	0.42	0.49	0.54

The values are shown with downward (↓) or level (↔) arrows, if decreased or equal in comparison to default docking scoring [10]. The values are underlined, if equal or improved in comparison to the equivalent BR-NIB results (i.e., no 3D ligand data included) [10]. Values are shown in bold and italics, if improved or equal to LBR-NiB (Table 1). ^(1)^ Training/test set ratios (70:30): the percentage of ligands used in the training (70%) in relation to the percentage used in the testing (30%); ^(2)^ Methods: flexible docking and solvent-enhanced BR-NiB either with the equal shape/ESP (0.5/0.5) weight or the shape only (1.0/0.0).

## Data Availability

The data presented in this study are available in supplementary materials.

## Supplementary Materials

The following supporting information can be downloaded at: https://www.mdpi.com/article/10.3390/ijms23147871/s1.
