# Supplementary material for: Ligand-Enhanced Negative Images Optimized for Docking Rescoring
Source: Int J Mol Sci. 2022 Jul 17;23(14):7871. doi: 10.3390/ijms23147871 (PMC9323918; doi:10.3390/ijms23147871)
Supplement: Supplementary file 1 [file ijms-23-07871-s001.zip › Supporting Information.pdf]

Supporting Information for

Ligand-Enhanced Negative Images Optimized for Docking Rescoring

Sami T. Kurkinen[1,2,3], Jukka V. Lehtonen[4,5], Olli T. Pentikäinen[1,2,3], Pekka A. Postila[1,2,3*]

[1] Institute of Biomedicine, Integrative Physiology and Pharmacy, University of Turku, FI-20014 Turku, Finland

[2] Aurlide Ltd., FI-21420 Lieto, Finland

[3] InFLAMES Research Flagship Center, University of Turku, FI-20014 Turku, Finland

[4] Structural Bioinformatics Laboratory, Biochemistry, Faculty of Science and Engineering, Åbo Akademi University, Turku, Finland

[5] InFLAMES Research Flagship Center, Åbo Akademi University, FI-20014 Turku, Finland

* Corresponding author: pekka.postila@utu.fi

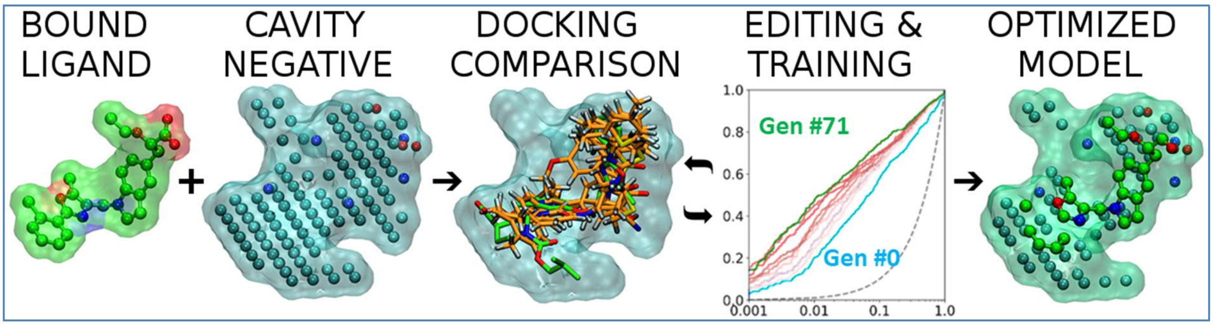

Table S1. Target protein structures, co-crystallized ligands, and benchmark sets.

**Target protein** [(1)]	**PDB code**	**Resolution (Å)**	**Co-crystallized ligand**	**Ratio** [(5)]	**No. of ligs** [(4)]	**No. of decs** [(5,6)]
COX2	3LN1	2.40	CEL	100:100<br>70:30<br>10:90	435/0<br>307/128<br>61/374	23,136/0<br>16,195/6941<br>2314/20,822
RXRα	1MV9 / 1MVC	1.90	HXA, BM6	100:100<br>70:30<br>10:90	131/0<br>91/40<br>13/118	6935/0<br>4855/2080<br>694/6241
MR	2AA2	1.95	AS4	100:100<br>70:30<br>10:90	94/0<br>65/29<br>9/85	5146/0<br>3602/1544<br>515/4631
NEU	1B9V	2.35	RA2	100:100<br>70:30<br>10:90	98/0<br>68/30<br>9/89	6197/0<br>4338/1859<br>620/5577
PDE5	1UDT / 1XOZ [(2)]	2.30 / 1.37	VIA, CIA	100:100<br>70:30<br>10:90	398/0<br>278/120<br>39/359	27,520/0<br>19,264/8256<br>2752/24,768
ER	1SJ0	1.90	E4D	100:100<br>70:30<br>10:90	383/0<br>273/110<br>38/345	20,663/0<br>14,464/6199<br>2066/18,597
PPARγ	2GTK	2.10	208	100:100<br>70:30<br>10:90	484/0<br>338/146<br>48/436	25,256/0<br>17,679/7577<br>2526/22,730
S1P1	3V2Y	2.80	ML5	100:100	30	15,000
PKA	4UJ1	1.77	NVX			
Rho-K2	7JNT	2.21	VFA			
HIV	3LP1	2.23	LP8			
HSP90	6LTK	2.14	E0G			
FAK	3BZ3	2.20	YAM			
Cath G	1T32	1.85	OHH [(6)]			
FXIa	7MBO	0.92	YXG			
FXIIa	6B77	2.37	CWV			

[(1)] Target proteins: cyclooxygenase 2 (COX2), retinoid X receptor alpha (RXRα), mineralocorticoid receptor (MR), neuraminidase (NEU), phosphodiesterase 5 (PDE5), estrogen receptor (ER), peroxisome proliferator-activated receptor gamma (PPARγ), sphingosine-1-phosphate receptor 1 (S1P1), protein kinase A (PKA), Rho-associated protein kinase 2 (Rho-K2), human immunodeficiency virus reverse transcriptase RNase (HIV), heat shock protein 90 (HSP90), focal adhesion kinase (FAK), Cathepsin G (Cath G), coagulation factor XIa (FXIa) and coagulation factor XIIa (FXIIa). [(2)] Only PDB entry 1UDT was used for the docking; however, both 1UDT and 1XOZ were used in the negative image-based (NIB) model generation. [(3)] Training/test set ratios (100:100, 70:30, 10:90): the percentage of ligands used in the training (100 %, 70 %, 10 %) in relation to the percentage used in the testing (100 %, 30 %, 90 %). [(4)] The number (No.) of active ligands (ligs) and decoy molecules (decs) after performing the ligand preparation with LIGPREP in MAESTRO (status before the docking screening). The source of the used benchmark test sets was DUD-E (A Database of Useful (Docking) Decoys –Enhanced) [12]. [(5)] The actual decoy numbers can be marginally lower, because the decoy sets contain duplicates and LIGPREP occasionally skips problematic compounds during SMILES-3D conversion. [(6)] The parts of the ligand that were outside the NIB model were excluded.

Table S2. Generation #0: Docking rescoring with the non-optimized models in training.

Train/test [(1)]	Method [(2)]	Yield	COX2 + CEL	RXRα + HXA	RXRα + BM6	MR + AS4	NEU + RA2	PDE5 + VIA/CIA [(3)]	ER + E4D	PPARγ + 208
100:100	LBR-NiB / Gen #0	AUC	0.72±0.01	0.81±0.02	0.88±0.02	0.82±0.03	0.86±0.02 (↔)	0.73±0.01 (↓)	0.67±0.02 (↓)	0.80±0.01 (↓)
		EFd 1%	1.8 (↓)	1.5 (↓)	9.9 (↓)	8.5	5.1	2.5 (↓)	13.8 (↓)	20.2 (↓)
		EFd 5%	6.4 (↓)	0.21 (↓)	40.4	34.1	28.6 (↓)	17.6 (↓)	29.2 (↓)	44.8 (↓)
		BR20	0.09 (↓)	19.1 (↓)	0.38	0.31	0.27 (↓)	0.16 (↓)	0.29 (↓)	0.42 (↓)
	LBR-NiB / Gen #0 + shape only	AUC	0.78±0.01	0.79±0.02 (↔)	0.85±0.02	0.75±0.03	0.91±0.02	0.73±0.01 (↓)	0.67±0.02 (↓)	0.77±0.01 (↓)
		EFd 1%	12.9	4.6 (↓)	25.2	12.8	9.2	2.3 (↓)	13.8 (↓)	8.7 (↓)
		EFd 5%	25.7	22.9 (↓)	45.0	29.8	51.0	17.8 (↓)	29.2 (↓)	31.2 (↓)
		BR20	0.29	0.23 (↓)	0.43	0.29	0.42	0.17 (↓)	0.29 (↓)	0.30 (↓)
70:30	LBR-NiB / Gen #0	AUC	0.72±0.02	0.80±0.03 (↔)	0.87±0.02	0.81±0.03	0.86±0.03 (↓)	0.66±0.02 (↓)	0.66±0.02 (↓)	0.80±0.01 (↓)
		EFd 1%	1.6 (↓)	1.1 (↓)	9.9 (↓)	6.2 (↓)	2.9 (↓)	2.1 (↓)	13.2 (↓)	21.0 (↓)
		EFd 5%	6.5 (↓)	17.6 (↓)	38.5	33.8	29.4 (↓)	7.6 (↓)	27.5 (↓)	45.9 (↓)
		BR20	0.09 (↓)	0.20 (↓)	0.38	0.30	0.26 (↓)	0.10 (↓)	0.28 (↓)	0.42 (↓)
70:30	LBR-NiB / Gen #0 + shape only	AUC	0.78±0.02	0.79±0.03 (↔)	0.84±0.02	0.74±0.04	0.91±0.02 (↔)	0.70±0.02 (↓)	0.66±0.02 (↓)	0.77±0.02 (↓)
		EFd 1%	12.1	5.5 (↓)	24.2	9.2	8.8	1.4 (↓)	13.2 (↓)	10.1 (↓)
		EFd 5%	24.8	23.1 (↓)	40.7	27.7	50.0	10.8 (↓)	27.5 (↓)	32.0 (↓)
		BR20	0.28	0.22 (↓)	0.41	0.26	0.41	0.12 (↓)	0.28 (↓)	0.30 (↓)
10:90	LBR-NiB / Gen #0	AUC	0.71±0.04 (↔)	0.70±0.08 (↔)	0.83±0.07 (↔)	0.78±0.09	0.87±0.08 (↔)	0.68±0.05 (↓)	0.74±0.05 (↔)	0.79±0.04 (↔)
		EFd 1%	6.6 (↓)	0.0 (↔)	0.0 (↔)	11.1	0.0 (↔)	5.1 (↓)	15.8	27.1 (↔)
		EFd 5%	9.8 (↓)	0.0 (↓)	23.1	22.2 (↔)	44.4 (↔)	7.7 (↓)	28.9 (↔)	47.9 (↓)
		BR20	0.14 (↓)	0.05 (↓)	0.23	0.26	0.32 (↓)	0.14 (↓)	0.32 (↓)	0.44 (↓)
10:90	LBR-NiB / Gen #0 + shape only	AUC	0.78±0.04	0.69±0.08 (↔)	0.78±0.08 (↔)	0.76±0.09	0.92±0.06 (↔)	0.75±0.04 (↔)	0.74±0.05 (↔)	0.77±0.04 (↔)
		EFd 1%	11.5	0.0 (↔)	0.0 (↔)	11.1	11.1	0.0 (↓)	15.8	10.4 (↓)
		EFd 5%	26.2	0.0 (↓)	30.8	33.3	44.4 (↔)	15.4 (↓)	28.9 (↔)	39.6 (↓)
		BR20	0.29	0.06 (↓)	0.28	0.30	0.52	0.14 (↓)	0.31 (↓)	0.35 (↓)

The values are shown with downward (↓) or level (↔) arrows, if decreased or equal in comparison to default docking scoring[10]. The values are underlined, if improved in comparison to the regular R-NiB, performed with the NIB model lacking 3D ligand data[10].

[(1)] Training/test set ratios (100:100, 70:30, 10:90): the percentage of ligands used in the training (100 %, 70 %, 10 %) in relation to the percentage used in the testing (100 %, 30 %, 90 %). The relevant training or test set percentage is underlined in the ratio column. [(2)] Methods: ligand-enhanced brute force negative image-based optimization (LBR-NiB; Figs. 2-3) for generation #0 (or Gen #0) either with the equal shape/ESP (0.5/0.5) weight or the shape only (1.0/0.0). Given that no hybrid model optimization has been performed yet at Gen #0 of LBR-NiB, the results are equivalent to R-NiB that relies only on the input NIB model[10]. [(3)] 70:30 and 10:90 training/test set ratios for PDE5 could not be compared to the regular R-NiB approach (no underlining).

Table S3. Generation #0: Docking rescoring with the non-optimized models in testing.

**Train/test** (1)	**Method** (2)	**Yield**	**COX2 + CEL**	**RXRα + HXA**	**RXRα + BM6**	**MR + AS4**	**NEU + RA2**	**PDE5 + VIA/CIA** (3)	**ER + E4D**	**PPARγ + 208**
70:30	LBR-NiB / Gen #0	AUC	0.73±0.02	0.82±0.04 (↔)	0.90±0.03	0.82±0.05	0.87±0.04 (↔)	0.67±0.03 (↓)	0.70±0.03 (↓)	0.79±0.02 (↓)
		EFd 1%	2.2 (↓)	0.0 (↓)	10.0 (↓)	13.8	10.0	1.7 (↓)	5.1 (↓)	16.4 (↓)
		EFd 5%	5.9 (↓)	22.5 (↓)	50.0	31.0	26.7 (↓)	11.7 (↓)	23.1 (↓)	43.8 (↓)
		BR20	0.10 (↓)	0.22 (↓)	0.42	0.32	0.29 (↔)	0.13 (↓)	0.23 (↓)	0.42 (↓)
70:30	LBR-NiB / Gen #0 + shape only	AUC	0.78±0.02	0.81±0.04 (↔)	0.87±0.04	0.77±0.05	0.90±0.04 (↔)	0.75±0.03 (↔)	0.72±0.03 (↔)	0.77±0.02 (↓)
		EFd 1%	28.1	5.0 (↓)	27.5	17.2	10.0	2.5 (↓)	22.2 (↓)	6.8 (↓)
		EFd 5%	45.2	22.5 (↓)	52.5	34.5	60.0	18.3 (↓)	37.6	29.5 (↓)
		BR20	0.32	0.23 (↓)	0.46	0.35	0.44	0.20 (↓)	0.38 (↓)	0.28 (↓)
10:90	LBR-NiB / Gen #0	AUC	0.72±0.01	0.82±0.02 (↔)	0.89±0.02	0.82±0.03	0.86±0.03 (↔)	0.66±0.02 (↓)	0.67±0.02 (↓)	0.80±0.01 (↓)
		EFd 1%	1.3	1.7 (↓)	11.0 (↓)	8.2	5.6	2.2 (↓)	13.3 (↓)	19.5 (↓)
		EFd 5%	6.1 (↓)	21.2 (↓)	46.6	36.4	28.1 (↓)	8.9 (↓)	29.1 (↓)	44.5 (↓)
		BR20	0.09 (↓)	0.23 (↓)	0.42	0.31	0.26 (↓)	0.10 (↓)	0.28 (↓)	0.41 (↓)
10:90	LBR-NiB / Gen #0 + shape only	AUC	0.78±0.01	0.81±0.02 (↔)	0.85±0.02	0.75±0.03	0.91±0.02	0.71±0.02 (↓)	0.67±0.02 (↓)	0.77±0.01 (↓)
		EFd 1%	12.8	5.9 (↓)	27.1	12.9	9.0	1.9 (↓)	17.9 (↓)	8.7 (↓)
		EFd 5%	25.3	25.4 (↓)	46.6	28.2	51.7	13.4 (↓)	32.3 (↓)	30.3 (↓)
		BR20	0.28	0.24 (↓)	0.45	0.29	0.41	0.15 (↓)	0.33 (↓)	0.29 (↓)

The values are shown with downward (↓) or level (↔) arrows, if decreased or equal in comparison to default docking scoring[10]. The values are underlined, if improved in comparison to the regular R-NiB, performed with the NIB model lacking 3D ligand data [10].

(1) Training/test set ratios (100:100, 70:30, 10:90): the percentage of ligands used in the training (100 %, 70 %, 10 %) in relation to the percentage used in the testing (100 %, 30 %, 90 %). The relevant training or test set percentage is underlined in the ratio column. (2) Methods: ligand-enhanced brute force negative image-based optimization (LBR-NiB; Figs. 2-3) for generation #0 (or Gen #0) either with the equal shape/ESP (0.5/0.5) weight or the shape only (1.0/0.0). Given that no hybrid model optimization has been performed yet at Gen #0 of LBR-NiB, the results are equivalent to R-NiB that relies only on the input NIB model [10]. (3) 70:30 and 10:90 training/test set ratios for PDE5 could not be compared to the regular R-NiB approach (no underlining).

Table S4. Ligand-enhanced brute force negative image-based optimization using cavity models combined with co-crystallized ligands in training.

**Train/ test** (1)	**Method** (2)	**Yield**	**COX2 + CEL**	**RXRα + HXA**	**RXRα + BM6**	**MR + AS4**	**NEU + RA2**	**PDE5 + VIA/CIA**	**ER + E4D**	**PPARγ + 208**
70:30	LBR-NiB	AUC	0.84±0.01	0.91±0.02	0.92±0.02	0.87±0.03	0.98±0.01	0.86±0.01	0.75±0.02	0.88±0.01
		EFd 1%	45.3	67.0	71.4	24.6	86.8	28.4	37.7	48.5
		EFd 5%	63.5	75.8	83.5	53.8	94.1	54.3	53.5	64.5
		BR20	0.61	0.75	0.81	0.50	0.92	0.49	0.53	0.63
	LBR-NiB + shape only	AUC	0.84±0.01	0.90±0.02	0.91±0.02	0.82±0.03	0.98±0.01	0.87±0.01	0.67±0.02	0.85±0.01
		EFd 1%	51.1	61.5	70.3	32.3	89.7	29.9	35.9	44.1
		EFd 5%	64.8	74.7	81.3	47.7	92.7	53.6	43.9	59.2
		BR20	0.63	0.73	0.80	0.46	0.92	0.49	0.45	0.59
10:90	LBR-NiB	AUC	0.83±0.03	0.91±0.06	0.93±0.05	0.78±0.09	1.00±0.01	0.88±0.04	0.78+0.04	0.88±0.03
		EFd 1%	47.5	61.5	84.6	55.6	88.9	38.4	55.3	58.3
		EFd 5%	63.9	76.9	84.6	55.6	100.0	69.2	57.9	70.8
		BR20	0.63	0.76	0.86	0.60	0.96	0.61	0.60	0.71
	LBR-NiB + shape only	AUC	0.83±0.03	0.82±0.07	0.93±0.05	0.79±0.09	1.00±0.01	0.87±0.04	0.82±0.04	0.84±0.04
		EFd 1%	52.5	69.2	84.6	55.6	100.0	56.4	57.9	58.3
		EFd 5%	63.9	76.9	92.3	55.6	100.0	74.4	60.5	68.8
		BR20	0.67	0.79	0.91	0.59	0.97	0.67	0.63	0.68

The rescoring values are shown in bold and italics, if improved in comparison to the docking of each training (70 %, 10 %) or test (30 % or 90 %) set. Only those AUC values that are within the error margin are highlighted. The underlined values are equal or better than the equivalent BR-NiB results [10].

(1) Training/test set ratios (100/100, 70/30, 10/90): the percentage of ligands used in the training (100 %, 70 %, 10 %) in relation to the percentage used in the testing (100 %, 30 %, 90 %). (2) Methods: flexible docking and LBR-NiB either with the equal shape/ESP (0.5/0.5) weight or the shape only (1.0/0.0).

Table S5. Ligand-enhanced brute force negative image-based optimization versus brute force negative image-based optimization in testing.

**Train/ test** (1)	**Method** (2)	**Yield**	**COX2 + CEL**	**RXRα + HXA**	**RXRα + BM6**	**MR + AS4**	**NEU + RA2**	**PDE5 + VIA/CIA**	**ER + E4D**	**PPARγ + 208**
100:100	LBR-NiB	EFd 1%	***12.6***	-12.2	***0.70***	-5.3	-1.1	***8.5***	-5.1	***21.1***
		EFd 5%	***11.3***	-14.5	-6.10	***8.6***	***1.1***	***12.8***	-2.9	***13.2***
		BR20	***0.11***	-0.1	-0.03	***0.02***	***0.01***	***0.09***	-0.05	***0.16***
	LBR-NiB + shape only	EFd 1%	***12.1***	***19.1***	***21.4***	0.0	***4.0***	***1.8***	***2.9***	***4.0***
		EFd 5%	***6.9***	***6.1***	***12.9***	***8.5***	***6.1***	***2.5***	***1.3***	***1.2***
		BR20	***0.06***	***0.1***	***0.16***	***0.05***	***0.05***	***0.03***	***0.02***	***0.01***
70:30	LBR-NiB	EFd 1%	***7.1***	-12.5	-10.0	0.0	***13.3***	***2.5***	-0.6	**13.7**
		EFd 5%	***12.3***	-12.5	-5.0	***6.9***	***10***	***15***	-6.8	**14.4**
		BR20	***0.1***	-0.05	-0.02	***0.06***	***0.09***	***0.11***	-0.02	**0.15**
	LBR-NiB + shape only	EFd 1%	-5.6	-20.0	***22.5***	-6.9	***3.3***	***9.2***	***10.3***	0.0
		EFd 5%	***3.0***	-7.5	***15.0***	***3.5***	-3.3	***12.5***	-0.3	***2.1***
		BR20	-0.01	-0.1	***0.14***	-0.02	***0.03***	***0.08***	***0.03***	***0.01***
10:90	LBR-NiB	EFd 1%	***5.0***	-30.5	-15.2	***8.2***	-7.9	***11.4***	-4.8	***23.9***
		EFd 5%	***7.4***	-26.5	-9.3	-2.3	-2.3	***18.1***	-3.7	***15.4***
		BR20	***0.07***	-0.2	-0.09	***0.02***	-0.03	***0.16***	-0.03	***0.18***
	LBR-NiB + shape only	EFd 1%	***5.6***	-0.8	***39.0***	***4.7***	***1.2***	***3.0***	-1.9	***1.8***
		EFd 5%	***2.2***	***1.7***	***41.6***	***3.5***	***2.2***	***0.9***	-5.7	***9.8***
		BR20	***0.04***	***0.03***	***0.37***	***0.03***	***0.03***	***0.03***	-0.06	***0.06***

The values are positive and shown in bold and italics, if improved in comparison to BR-NiB (100 %, 30 % or 90 %). The negative values (designated by minus signs) indicate cases where the LBR-NiB (Table 1) generated weaker result than BR-NiB[10]. The AUC value comparison is omitted.

(1) Training/test set ratios (100/100, 70/30, 10/90): the percentage of ligands used in the training (100 %, 70 %, 10 %) in relation to the percentage used in the testing (100 %, 30 %, 90 %). (2) Methods: flexible docking and LBR-NiB either with the equal shape/ESP (0.5/0.5) weight or the shape only (1.0/0.0).

Table S6. Ligand-enhanced brute force negative image-based optimization of preoptimized cavity models combined with co-crystallized ligands in training.

**Train/ test** (1)	**Method** (2)	**Yield**	**COX2 + CEL**	**RXRα + HXA**	**RXRα + BM6**	**MR + AS4**	**NEU + RA2**	**PDE5 + VIA/CIA**	**ER + E4D**	**PPARγ + 208**
70:30	BR-NiB + LBR-NiB	AUC	0.80±0.02	0.91±0.02	0.96±0.01	0.85±0.03	0.98±0.01	0.85±0.01	0.82±0.02 (↔)	0.88±0.02 (↔)
		EFd 1%	51.1	67.0	72.5	33.8	83.8	25.2	34.1	39.9
		EFd 5%	61.6	74.7	84.6	53.8	91.2	49.3	50.5	63.9
		BR20	0.62	0.75	0.82	0.50	0.90	0.44	0.49	0.60
	BR-NiB + LBR-NiB + shape only	AUC	0.84±0.02	0.89±0.02	0.91±0.02	0.83±0.03	0.98±0.01	0.87±0.01	0.64±0.02 (↓)	0.85±0.01 (↔)
		EFd 1%	51.5	67.0	72.5	30.8	85.3	29.5	35.9	42.0
		EFd 5%	61.6	72.5	79.1	49.2	94.1	55.0	43.6	58.6
		BR20	0.64	0.74	0.80	0.48	0.91	0.49	0.45	0.58
10:90	BR-NiB + LBR-NiB	AUC	0.83±0.03	0.90±0.06	0.91±0.05	0.85±0.08	1.00±0.01	0.89±0.03 (↔)	0.76±0.05	0.88±0.03
		EFd 1%	44.3	53.8	84.6	66.7	100.0	61.5	55.3	45.8
		EFd 5%	63.9	84.6	84.6	66.7	100.0	69.2	55.3	62.5
		BR20	0.64	0.79	0.86	0.69	0.98	0.64	0.58	0.62
	BR-NiB + LBR-NiB + shape only	AUC	0.83±0.03	0.81±0.07	0.92±0.05	0.82±0.03	1.00±0.00	0.89±0.03 (↔)	0.84±0.04	0.83±0.04
		EFd 1%	54.1	61.5	76.9	23.9	100.0	51.3	55.3	52.1
		EFd 5%	65.6	76.9	92.3	39.8	100.0	66.7	68.4	58.3
		BR20	0.67	0.77	0.88	0.39	1.0	0.62	0.67	0.60

The values are shown with downward (↓) or level (↔) arrows, if decreased or equal in comparison to default docking scoring[10]. The values are underlined, if equal or improved in comparison to the equivalent BR-NIB results (*i.e.*, no 3D ligand data included)[10]. Values are shown in bold and italics, if improved or equal to LBR-NiB performed with the co-crystallized ligands and non-optimized NIB models (Table 1).

(1) Training/test set ratios (100/100, 70/30, 10/90): the percentage of ligands used in the training (100 %, 70 %, 10 %) in relation to the percentage used in the testing (100 %, 30 %, 90 %). (2) Methods: flexible docking and LBR-NiB either with the equal shape/ESP (0.5/0.5) weight or the shape only (1.0/0.0).

Table S7. Ligand-enhanced brute force negative image-based optimization of preoptimized cavity models versus brute force negative image-based optimization in testing.

**Train/ test** (1)	**Method** (2)	**Yield**	**COX2 + CEL**	**RXRα + HXA**	**RXRα + BM6**	**MR + AS4**	**NEU + RA2**	**PDE5 + VIA/CIA**	**ER + E4D**	**PPARγ + 208**
100:100	LBR-NiB	EFd 1%	***10.8***	-7.7	-2.3	-2.1	***3.0***	***5.3***	-1.7	***12.6***
		EFd 5%	***7.3***	-10.7	-7.6	***5.4***	0.0	***9.8***	-2.6	***8.7***
		BR20	***0.09***	-0.08	-0.04	***0.02***	***0.01***	***0.07***	-0.03	***0.11***
	LBR-NiB + shape only	EFd 1%	***11.8***	***17.5***	***25.2***	***1.0***	***6.1***	***3.8***	***3.7***	***4.6***
		EFd 5%	***6.2***	***6.8***	***11.4***	***5.3***	***4.0***	***4.5***	***3.4***	***0.6***
		BR20	***0.06***	***0.1***	***0.16***	***0.04***	***0.05***	***0.04***	***0.03***	***0.01***
70:30	LBR-NiB	EFd 1%	***12.2***	-12.5	-2.5	0.0	***6.6***	***5.0***	-7.4	***8.2***
		EFd 5%	***7.1***	-7.5	-7.5	***6.9***	***6.6***	***8.3***	-10.2	***13.0***
		BR20	***0.08***	-0.04	-0.02	***0.07***	***0.05***	***0.08***	-0.07	***0.11***
	LBR-NiB + shape only	EFd 1%	***10.0***	***20.0***	***25.0***	-17.8	***16.6***	***2.5***	***10.3***	***2.0***
		EFd 5%	***5.2***	***7.5***	***15.0***	***3.5***	***10.0***	***9.1***	***2.3***	***0.7***
		BR20	***0.07***	***0.08***	***0.15***	-0.03	***0.02***	***0.05***	***0.04***	***0.02***
10:90	LBR-NiB	EFd 1%	***2.2***	-23.7	-6.7	***12.1***	***8.7***	***13.9***	-4.9	***22.0***
		EFd 5%	***6.6***	-16.9	-11	***1.0***	***9.8***	***20.4***	-2.5	***20.0***
		BR20	***0.05***	-0.14	-0.05	***0.05***	***0.09***	***0.17***	-0.03	***0.19***
	LBR-NiB + shape only	EFd 1%	***7.1***	***17.8***	***33.9***	***6.2***	***12.3***	***2.6***	-0.9	***4.2***
		EFd 5%	***0.4***	***17.8***	***36.5***	***4.5***	***12.2***	***4.3***	-0.1	***11.2***
		BR20	***0.04***	***0.17***	***0.34***	***0.04***	***0.12***	***0.05***	-0.01	***0.08***

The values are positive and shown in bold and italics, if improved in comparison to BR-NiB (100 %, 30 % or 90 %). The negative values (designated by minus signs) indicate cases where the LBR-NiB (Table 1) generated weaker result than BR-NiB[10]. The AUC value comparison is omitted.

(1) Training/test set ratios (100/100, 70/30, 10/90): the percentage of ligands used in the training (100 %, 70 %, 10 %) in relation to the percentage used in the testing (100 %, 30 %, 90 %). (2) Methods: flexible docking and LBR-NiB either with the equal shape/ESP (0.5/0.5) weight or the shape only (1.0/0.0).

Table S8. Ligand-enhanced brute force negative image-based optimization with the Maximum Unbiased Validation set.

Method [(1)]	Yield	S1P1	PKA	Rho-K2	HIV	HSP90	FAK	CathG	FXIa	FXIIa
Docking	AUC	0.53±0.05	0.54±0.05	0.44±0.05	0.45±0.05	0.42±0.05	0.46±0.05	0.38±0.05	0.38±0.05	0.32±0.04
	EFd 0.5%	3.3	0.0	0.0	3.3	0.0	3.3	0.0	0.0	0.0
	EFd 1%	3.3	0.0	3.3	3.3	0.0	3.3	0.0	0.0	0.0
	EFd 5%	10.0	6.7	6.7	3.3	0.0	6.7	3.3	0.0	0.0
	BR20	0.08	0.03	0.06	0.05	0.00	0.05	0.02	0.04	0.00
BR-NiB	AUC	0.60±0.05	***0.75±0.05***	***0.71±0.05***	***0.69±0.05***	0.49±0.05	0.53±0.05	***0.65±0.05***	***0.70±0.05***	***0.69±0.05***
	EFd 0.5%	***26.7***	***6.7***	***10.0***	***6.7***	***6.7***	***10.0***	***10.0***	***16.7***	***10.0***
	EFd 1%	***26.7***	***13.3***	***33.3***	***23.3***	***23.3***	***23.3***	***23.3***	***26.7***	***16.7***
	EFd 5%	***26.7***	***46.7***	***43.3***	***40.0***	***26.7***	***23.3***	***40.0***	***43.3***	***33.3***
	BR20	***0.27***	***0.35***	***0.37***	***0.33***	***0.24***	***0.22***	***0.33***	***0.36***	***0.27***
LBR-NiB	AUC	0.63±0.05	0.57±0.05	0.47±0.05	***0.64±0.05***	***0.53±0.05***	0.56±0.05	***0.56±0.05***	0.35±0.04	***0.70±0.05***
	EFd 0.5%	3.3	***10.0***	***10.0***	***10.0***	***6.7***	***13.3***	***20.0***	0.0	***6.7***
	EFd 1%	***3.3***	***16.7***	***23.3***	***13.3***	***13.3***	***13.3***	***20.0***	0.0	***10.0***
	EFd 5%	***20.0***	***23.3***	***23.3***	***46.7***	***33.3***	***26.7***	***23.3***	***10.0***	***30.0***
	BR20	***0.15***	***0.21***	***0.22***	***0.32***	***0.25***	***0.23***	***0.22***	***0.07***	***0.24***
BR-NiB + LBR-NiB	AUC	0.57±0.05	***0.68±0.05***	0.45±0.05	***0.62±0.05***	***0.56±0.05***	0.54±0.05	***0.54±0.05***	***0.68±0.05***	***0.69±0.05***
	EFd 0.5%	***16.7***	***6.7***	***6.7***	***13.3***	***13.3***	***6.7***	***20.0***	***10.0***	***6.7***
	EFd 1%	***16.7***	***20.0***	***13.3***	***16.7***	***20.0***	***10.0***	***30.0***	***16.7***	***10.0***
	EFd 5%	***16.7***	***36.7***	***30.0***	***30.0***	***26.7***	***26.7***	***36.7***	***26.7***	***23.3***
	BR20	***0.16***	***0.31***	***0.23***	***0.28***	***0.23***	***0.19***	***0.33***	***0.24***	***0.21***

The values are shown in bold and italics, if improved over docking. The best values are underlined and the AUC values within the error margin were considered equal in this comparison. No training/test (100:200) set division was applied with the MUV sets. The results for docking and BR-NiB (not shape only) were calculated in a prior study[10]. See PDB codes and target full names from the Table S1.

[(1)] Methods: flexible docking and LBR-NiB with the equal shape/ESP (0.5/0.5) weigh in scoring or stepwise BR-NiB+LBR-NiB processing with the equal shape/ESP scoring.

Table S9. Ligand-enhanced brute force negative image-based optimization using shape only with the Maximum Unbiased Validation set.

Method [1]	Yield	S1P1	PKA	Rho-K2	HIV	HSP90	FAK	CathG	FXIa	FXIIa
Docking	AUC	0.53±0.05	0.54±0.05	0.44±0.05	0.45±0.05	0.42±0.05	0.46±0.05	0.38±0.05	0.38±0.05	0.32±0.04
	EFd 0.5%	3.3	0.0	0.0	3.3	0.0	3.3	0.0	0.0	0.0
	EFd 1%	3.3	0.0	3.3	3.3	0.0	3.3	0.0	0.0	0.0
	EFd 5%	10.0	6.7	6.7	3.3	0.0	6.7	3.3	0.0	0.0
	BR20	0.08	0.03	0.06	0.05	0.00	0.05	0.02	0.04	0.00
BR-NiB + shape only	AUC	0.60±0.05	***0.68±0.05***	0.44±0.05	***0.65±0.05***	0.48±0.05	0.52±0.05	***0.56±0.05***	***0.77±0.05***	***0.73±0.05***
	EFd 0.5%	***13.3***	***13.3***	***10.0***	***10.0***	***10.0***	***16.7***	***23.3***	***10.0***	***16.7***
	EFd 1%	***23.3***	***20.0***	***26.7***	***20.0***	***23.3***	***30.0***	***30.0***	***16.7***	***16.7***
	EFd 5%	***30.0***	***43.3***	***26.7***	***36.7***	***26.7***	***30.0***	***36.7***	***40.0***	***23.3***
	BR20	***0.27***	***0.35***	***0.25***	***0.33***	***0.24***	***0.28***	***0.34***	***0.37***	***0.25***
LBR-NiB + shape only	AUC	0.59±0.05	0.60±0.05	0.49±0.05	***0.64±0.05***	***0.56±0.05***	0.46±0.05	***0.57±0.05***	***0.73±0.05***	***0.72±0.05***
	EFd 0.5%	***16.7***	***10.0***	***13.3***	***6.7***	***6.7***	***13.3***	***30.0***	***3.3***	***10.0***
	EFd 1%	***23.3***	***16.7***	***20.0***	***13.3***	***13.3***	***20.0***	***36.7***	***16.7***	***13.3***
	EFd 5%	***33.3***	***40.0***	***26.7***	***43.3***	***33.3***	***23.3***	***36.7***	***46.7***	***33.3***
	BR20	***0.28***	***0.32***	***0.24***	***0.31***	***0.28***	***0.21***	***0.35***	***0.33***	***0.25***
BR-NiB + LBR-NiB + shape only	AUC	***0.65±0.05***	***0.67±0.05***	0.43±0.05	***0.65±0.05***	0.43±0.05	0.51±0.05	***0.56±0.05***	***0.76±0.05***	***0.73±0.05***
	EFd 0.5%	***10.0***	***13.3***	***10.0***	***6.7***	***3.3***	***23.3***	***23.3***	***3.3***	***10.0***
	EFd 1%	***23.3***	***20.0***	***20.0***	***13.3***	***3.3***	***30.0***	***26.7***	***13.3***	***16.7***
	EFd 5%	***33.3***	***50.0***	***26.7***	***43.3***	***13.3***	***30.0***	***36.7***	***53.3***	***23.3***
	BR20	***0.30***	***0.39***	***0.24***	***0.34***	***0.10***	***0.28***	***0.34***	***0.39***	***0.26***

The values are shown in bold and italics, if improved over docking. The best values are underlined and the AUC values within the error margin were considered equal in this comparison. No training/test (100:100) set division was applied with the MUV sets. The results for docking and BR-NiB (not shape only) were calculated in a prior study [10]. See PDB codes and target full names from the Table S1.

[1] Methods: flexible docking, LBR-NiB with the shape only scoring or stepwise BR-NiB+LBR-NiB processing with the shape only scoring.

Table S10. Ligand-enhanced brute force negative image-based optimization with cavity models combined with docked water in training.

**Train/ test** ([1])	**Method** ([2])	**Yield**	**COX2**	**RXRα**	**MR**	**NEU**	**PDE5**	**ER**	**PPARγ**
water 70:30	LBR-NiB	AUC	0.78±0.04	0.95±0.02	0.66±0.04	0.97±0.03	0.83±0.02	0.66±0.02	0.85±0.01
		EFd 1%	27.9	***82.5***	27.7	***88.2***	20.9	***42.1***	40.2
		EFd 5%	60.7	***87.5***	43.1	94.1	48.2	47.3	59.1
		BR20	0.56	***0.87***	0.43	0.91	0.43	0.49	0.57
	LBR-NiB + shape only	AUC	0.83±0.01	0.89±0.02	0.76±0.04	0.98±0.01	0.86±0.01	0.65±0.02	0.84±0.01
		EFd 1%	46.6	57.1	32.3	80.9	***33.1***	***37.7***	42.0
		EFd 5%	61.2	***75.8***	47.7	***94.1***	52.5	43.6	58.3
		BR20	0.61	0.72	***0.47***	0.90	***0.50***	***0.46***	0.57
ethanol 70:30	LBR-NiB	AUC	0.79±0.02	0.95±0.02	0.81±0.03	0.98±0.01	0.81±0.02	0.65±0.02	0.83±0.01
		EFd 1%	43.6	***84.6***	***35.4***	**89.7**	20.5	***40.3***	26.3
		EFd 5%	55.0	***87.9***	50.8	**97.1**	46.4	***47.3***	50.9
		BR20	0.56	***0.87***	0.48	**0.94**	0.42	***0.49***	0.47
	LBR-NiB + shape only	AUC	0.82±0.01	*0.89*±0.02	0.79±0.05	0.97±0.01	0.84±0.01	0.63±0.02	0.85±0.01
		EFd 1%	44.6	*50.5*	***33.8***	83.8	29.5	***37.0***	40.8
		EFd 5%	59.9	*73.6*	***53.8***	91.2	53.2	42.0	59.5
		BR20	0.59	*0.70*	***0.50***	0.89	0.48	0.45	0.58

The values are shown in bold and italics, if improved over LBR-NiB performed with the co-crystallized ligands with the training sets (70 %, 10 %) (Table 1). The values are underlined, if improved in comparison to the regular BR-NIB (*i.e.*, no 3D ligand data included) [10].

Table S11. The test set enrichment for the ligand-based rescoring.

Train/ test	Yield	COX2	RXRα		MR	NEU	PDE5		ER	PPARγ
			HXA	BM6			VIA	CIA		
100:100	AUC	***0.69±0.01***	0.26±0.02	0.28±0.02	***0.61±0.03***	0.50±0.03	0.53±0.01	0.61±0.02	0.56±0.02	0.32±0.01
	EFd 1%	***20.0***	2.3	2.3	***11.7***	3.1	6.8	9.3	5.2	2.5
	EFd 5%	***28.3***	4.6	3.1	***25.5***	11.2	10.6	20.6	11.2	6.8
	BR20	***0.30***	0.04	0.04	***0.25***	0.10	0.12	0.20	0.12	0.07
70:30	AUC	***0.67±0.03***	0.26±0.03	0.28±0.03	***0.66±0.06***	0.46±0.05	0.52±0.03	0.61±0.03	0.52±0.03	0.32±0.02
	EFd 1%	***20.7***	0.0	5.0	***10.3***	***3.3***	4.2	7.5	4.3	3.4
	EFd 5%	***28.1***	7.5	5.0	***34.5***	6.7	9.2	21.7	9.4	7.5
	BR20	***0.30***	0.06	0.06	***0.30***	0.07	0.10	0.19	0.09	0.07
10:90	AUC	***0.69±0.01***	0.27±0.02	0.29±0.02	***0.60±0.03***	0.48±0.03	0.53±0.02	0.61±0.02	0.56±0.02	0.32±0.01
	EFd 1%	***20.4***	1.7	2.5	***10.6***	***3.4***	6.7	9.2	4.3	2.8
	EFd 5%	***28.8***	5.1	3.4	***23.5***	11.2	10.9	20.9	11.2	7.3
	BR20	***0.31***	0.04	0.04	***0.25***	0.10	0.12	0.20	0.12	0.07

The values that are as good as or better than the docking scoring [10] are shown in bold and italics. In the ligand-based rescoring (PLANTS + X-ray/SHAEP), the docking poses are directly compared without geometry optimization in SHAEP to the ligand 3D conformers co-crystallized with the target proteins (Table S1). The testing was performed using equal shape/electrostatic potential (0.5/0.5) weight. The Wilcoxon statistic[40] was used for the AUC error estimation. Data excluding RXRα with BM6 ligand and PDE5 from a previous study (Table S9) [10].

Table S12. Tanimoto similarity analysis of top-ranked active ligands against the co-crystallized ligands incorporated into the hybrid models.

Target	Co-crystal ligand	Method	Co-crystal *vs.* Top 5 %	Co-crystal *vs.* All actives	Top 5 % vs. all actives	Different actives at top 5 %	Different actives at top 25 %
**COX2**	CEL	BR-NiB<br>LBR-NiB	0.224<br>0.255	0.081	0.143<br>0.174	45 %	27 %
**RXRα #1** [1]	HXA	BR-NiB<br>LBR-NiB	0.193<br>0.253	0.124	0.069<br>0.129	100 %	42 %
**RXRα #2** [1]	BM6	BR-NiB<br>LBR-NiB	0.016<br>0.015	0.016	0.000<br>-0.001	57 %	52 %
**MR**	AS4	BR-NiB<br>LBR-NiB	0.111<br>0.308	0.024	0.087<br>0.284	100 %	58 %
**NEU**	RA2	BR-NiB<br>LBR-NiB	0.027<br>0.021	0.037	-0.01<br>-0.016	100 %	60 %
**PDE5 #1** [1]	VIA	BR-NiB<br>LBR-NiB	0.066<br>0.049	0.071	-0.005<br>-0.022	65 %	43 %
**PDE5 #2** [1]	CIA	BR-NiB<br>LBR-NiB	0.129<br>0.063	0.089	0.04<br>-0.026	65 %	43 %
**ER**	E4D	BR-NiB<br>LBR-NiB	0.182<br>0.289	0.124	0.058<br>0.165	58 %	38 %
**PPARγ**	208	BR-NiB<br>LBR-NiB	0.117<br>0.108	0.073	0.044<br>0.035	96 %	59 %

LBR-NiB data calculated for Table 1 compounds. [1] With RXRα, two separate NIB models containing either HXA or BM6 crystallized ligand poses were used, whereas in the case of PDE5, the two crystallized ligands were included in the same NIB model.

Table S13. Root-Mean-Square Deviation analysis of the selected docking poses against the co-crystallized ligands.

	**LBR-NIB**			**BR-NiB + LBR-NIB**			**BR-NIB**			**Docking**			**All docking poses**			**X-ray**
**Target**	**< 1 Å**	**< 2 Å**	**< 3 Å**	**< 1 Å**	**< 2 Å**	**< 3 Å**	**< 1 Å**	**< 2 Å**	**< 3 Å**	**< 1 Å**	**< 2 Å**	**< 3 Å**	**< 1 Å**	**< 2 Å**	**< 3 Å**	**N/A**
**COX2**	1	1	2	**2**	**3**	**3**	**2**	2	2	**2**	**3**	**3**	4	7	8	8
**RXRα**	***3***	***4***	***6***	***2***	***3***	***6***	***3***	***4***	***6***	1	3	***6***	4	6	6	6
**MR**	***4***	4	4	***4***	***5***	***5***	2	2	2	***4***	***5***	***5***	4	5	5	5
**NEU**	***3***	***6***	***6***	***3***	***6***	***6***	2	5	***6***	1	3	4	3	7	8	8
**PDE5**	0	***1***	***1***	0	0	***1***	0	0	***1***	0	0	0	0	2	2	4
**ER**	0	2	3	0	2	3	1	3	***6***	0	***4***	***6***	1	8	8	10
**PPARγ**	***1***	1	3	0	0	1	0	1	1	0	***2***	***4***	1	4	9	17
**Total**	12	19	25	11	19	25	10	17	24	8	20	28	17	39	46	60
**% Correct**	70.6	48.7	54.3	64.7	48.7	54.3	58.8	43.6	52.2	47.1	51.3	60.9	28.3	65.0	76.7	100.0

The values are shown in bold and italics, if improved over docking. The best values are underlined and the AUC values within the error margin were considered equal in this comparison. The results for docking and BR-NiB were calculated in a prior study[10]. The representative PDB codes for the used X-ray crystal structures were the following: 4K6I, 1FM9, 1RDT, 1MVC, 4K4J and 3A9E for the RXRα; 4PH9, 4M11, 3QMO, 5KIR, 1PXX, 3NT1, 5JVZ and 4COX for the COX-2; 3TGE, 3HC8, 1XOZ and 1TBF for the PDE5; 5MWY, 3VHU, 2AA5, 2AA2 and 4UDA for the MR; 1B9V, 1XOG, 2QWE, 1A4Q, 2QWG, 1LTF and 6HCX for the NEU; 1L2I, 1ERR, 1XQC, 2IOK, 5TLU, 5WGD, 4XI3, 2IOG, 5KR9, 2I0G, 3ERD, 1X7R AND 3ERT for ER, and 3GBK, 6D8X, 2G0H, 2HWR, 2HFP, 2Q59, 2Q5S, 2Q8S, 2F4B, 3KMG, 2P4Y, 3BC5, 5Y2O, 3D6D, 2I4J, 2ATH, 6DGO and 1NYX for PPARγ.

The percentage values for all docking poses (10 poses / cmpd) were calculated by dividing the correctly sampled poses with the actual co-crystallized ligand number. The percentage values for the LBR-NiB, BR-NIB+LBR-NiB, BR-NIB and the default docking scoring of PLANTS were calculated by dividing the correctly selected pose number all docking poses number that had been sampled correctly by the docking algorithm.
